# Identification of epigenetic dysregulation gene markers and immune landscape in kidney renal clear cell carcinoma by comprehensive genomic analysis

**DOI:** 10.3389/fimmu.2022.901662

**Published:** 2022-08-18

**Authors:** Linli Xie, Shuang Wu, Rong He, Sisi Li, Xiaodan Lai, Zhe Wang

**Affiliations:** ^1^ Department of Pharmacy, Southwest Hospital, Third Military Medical University (Army Medical University), Chongqing, China; ^2^ Department of Oncology and Southwest Cancer Center, Southwest Hospital, Third Military Medical University (Army Medical University), Chongqing, China; ^3^ Institute of Pathology and Southwest Cancer Center, Southwest Hospital, Third Military Medical University (Army Medical University), Chongqing, China; ^4^ Department of Pharmacy, No. 958 Hospital of Chinese People's Liberation Army (PLA), Chongqing, China

**Keywords:** kidney cancer, multi-omics data, prognosis, histone-modified genes, epigenetic modification

## Abstract

Kidney cancer is one the most lethal cancers of the urinary system, but current treatments are limited and its prognosis is poor. This study focused on kidney renal clear cell carcinoma (KIRC) and analyzed the relationship between epigenetic alterations and KIRC prognosis, and explored the prognostic significance of these findings in KIRC patients. Based on multi-omics data, differentially expressed histone-modified genes were identified using the R package limma package. Gene enhancers were detected from data in the FANTOM5 database. Gene promoters were screened using the R package ChIPseeker, and the Bumphunter in the R package CHAMP was applied to screen differentially methylated regions (DMR). Kyoto Encyclopedia of Genes and Genomes (KEGG) pathway analysis and Gene Ontology (GO) functional enrichment analysis of genes was performed using the R package clusterProfiler. We identified 51 dysregulated epigenetic protein coding genes (epi-PCGs) from 872 epi-PCGs, and categorized three molecular subtypes (C1, C2, and C3) of KIRC samples with significantly different prognosis. Notably, among the three molecular subtypes, we found a markedly differential immune features in immune checkpoints, cytokines, immune signatures, and immune cell distribution. C2 subtype had significantly lower enrichment score of IFNγ, cytotoxic score (CYT), and angiogenesis. In addition, an 8-gene signature containing 8 epi-PCGs (ETV4, SH2B3, FATE1, GRK5, MALL, HRH2, SEMA3G, and SLC10A6) was developed for predicting KIRC prognosis. Prognosis of patients with a high 8-gene signature score was significantly worse than those with a low 8-gene signature score, which was also validated by the independent validation data. The 8-gene signature had a better performance compared with previous signatures of KIRC. Overall, this study highlighted the important role of epigenetic regulation in KIRC development, and explored prognostic epi-PCGs, which may provide a guidance for exploiting further pathological mechanisms of KIRC and for developing novel drug targets.

## Introduction

Kidney cancer is the most lethal cancer of the urinary system, and shows an increasing incidence in recent years (1, 2). Due to a lack of specific clinical manifestations of kidney cancer, about 20-25% of patients have already developed distant metastasis by the time of diagnosis (3). For localized kidney cancer, local surgical resection in the form of partial or radical nephrectomy offers the possibility of partial cure. However, patients who have developed local recurrence or distant metastases are relatively resistant to conventional chemotherapy and radiotherapy and have a low 5-year survival rate (4, 5). Immunotherapy, especially immune checkpoint inhibitors, creates the hope for treating metastatic kidney cancer. For instance, monotherapy (nivolumab) or combined therapy (nivolumab and ipilimumab) shows favorable results on prolonged oval survival (6). Combined with other therapeutics such as tyrosine kinase inhibitors (TKIs), prolonged progression-free survival can be also realized in early phase trials (7). Nevertheless, a large proportion of kidney cancer patients still could not benefit from the immunotherapy due to individual differences. Therefore, to benefit more patients from immunotherapy, molecular subtyping may serve a role for assisting personalized therapies and reducing unnecessary treatment. So far, we face a lack of biomarkers for prognosis prediction and drug targets for therapeutic intervention, target-specific precision therapy for kidney cancer treatment, and KIRC patients often develop a poor prognosis. Therefore, there is an urgent need to find reliable new biomarkers to better understand the mechanisms of kidney cancer progression and to further develop new therapeutic strategies.

The essence of tumor occurrence and development is the inactivation of tumor suppressor genes and the activation of tumor-promoting genes. It takes a long time from the initial genetic change to evolve to a solid tumor. There is an epigenetic change prior to genetic change, or it is said that dysregulated gene expressions are caused by epigenetic changes. Studies have shown that epigenetic changes can regulate gene expression. Common epigenetic modifications include DNA methylation and acetylation, histone methylation and acetylation. Especially, histones modification, which commonly refers to methylation and acetylation, plays an important role in abnormal expression of genes. The modification of histone acetylation is based on the acetylation modification of histone lysine residues, which is largely related to transcriptional activation (8), and such a transcriptional activation is closely associated with the phenotype of a variety of tumors (9). When hyperacetylation occurs, particularly in proto-oncogenes, gene expression may be activated, and the hypoacetylation of tumor suppressor genes is usually located in the promoter, which will cause gene silencing when co-occurring with DNA methylation (10). The function of histone methylation modification is more complicated than that of histone acetylation modification in tumors (11, 12), but it is generally believed that the modification of histone methylation will reduce the transcription of target genes (13). Still, the relationship between such a modification and tumor development needs further research.

Current studies have shown that abnormal histone methylation is an independent prognostic marker of kidney cancer and a potential clinical target of kidney cancer (14–16). Various gene signatures related to epigenetic dysregulation have been developed for predicting the prognosis of renal cell carcinoma. For example, Zhou et al. analyzed the copy number variations (CNVs) of N6-methyladenosine (m6A) regulatory genes in clear cell renal cell carcinoma (ccRCC) samples, and observed a significant correlation between their CNVs and either overall survival or disease-free survival (17). Based on the expression of 19 m6A regulators, Zheng et al. constructed three molecular subtypes and established a seven-gene signature for ccRCC patients (18). Using two-way hierarchical clustering for methylation array data of ccRCC, three candidate genes with hypermethylation were identified and were significantly associated with metastatic free survival (19). However, limited studies comprehensively analyzed the epigenetic-dysregulated genes in kidney cancer, and less findings on the effect of epigenetic dysregulation on tumorigenesis and tumor pathology from different aspects such as tumor microenvironment and immune response were available.

Therefore, in this study, we focused on differential expressed genes and epigenetic-dysregulated genes concerning H3K27ac, H3K4me1, and H3K4me3, and identified 51 epigenetic protein-coding genes (epi-PCGs) associated with RCC prognosis. We constructed three molecular subtypes based on 51 epi-PCGs, and found significant differences on tumor microenvironment among the three subtypes. Finally, with the epi-PCGs, we constructed an 8-gene prognostic risk model that demonstrated a stable prediction performance in both the training set and the verification set. Our research results help better understand the abnormal epigenetic regulation of PCG expression in KIRC.

## Materials and methods

### Data download and preprocessing

**Figure 1:**
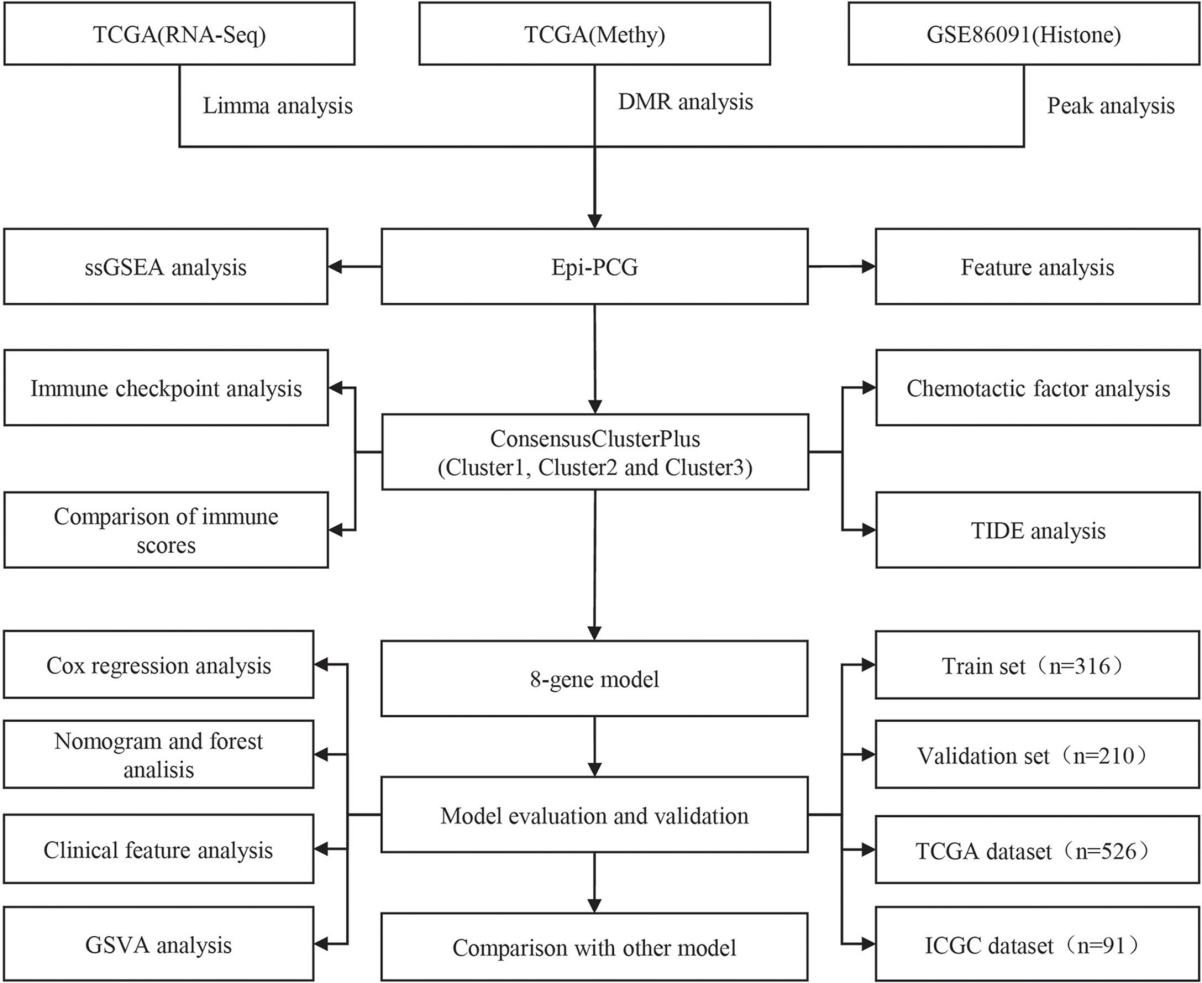
The work flow of this study.

The work flow of this study was shown in **Figure 1**. We downloaded the gene expression profile of kidney renal clear cell carcinoma (KIRC) and expression profile data such as fragments per kilobase million (FPKM), count number of normal samples, and clinical information of corresponding healthy control samples from the TCGA database (The Cancer Genome Atlas, https://www.cancer.gov/about-nci/organization/ccg/research/structural-genomics/tcga),and converted FPKM to TPM (transcript per million). Based on the gene annotation file of GENCODE, the expression profile was divided into long-non coding RNAs (lncRNAs) and positive correlation genes (PCGs), and we converted the Ensembl ID of these genes into Gene Symbol. At the same time, the RECA-EU data set with survival time was downloaded from the International Cancer Genome Consortium (ICGC) (https://dcc.icgc.org/) database. A total of 526 and 91 KIRC samples were included finally in TCGA and ICGC data sets respectively. See **Table 1** for the clinical information of the processed samples.

**Table 1:** Clinical information of the sample in TCGA and ICGC datasets.

Clinical Features	TCGA	ICGC
**OS**		
Alive	356	61
Dead	170	30
**Gender**		
Male	343	52
Female	183	39
**T Stage**		
T1	267	
T2	69	
T3	179	
T4	11	
**N Stage**		
N0	238	
N1	16	
NX	272	
**M Stage**		
M0	436	
M1	80	
MX	10	
**Stage**		
I	261	
II	57	
III	123	
IV	82	
X	3	
**Grade**		
G1	13	
G2	226	
G3	205	
G4	74	
GX	8	
**Age**		
>60	262	45
<=60	264	46

### The 450K methylation chip data and preprocessing

In this study, the KIRC chip data (HumanMethylation450 microarray) (20) was downloaded from TCGA database. According to the provided chip data, CpG with cross-reactive probes were removed. We further excluded the unstable methylation sites including CpG sites and single nucleotide sites locating in X/Y chromosome. Based on the sample number of KIRC, the chip data was split into 319 KIRC samples and 160 normal samples. The K-nearest neighbor (KNN) method (21), which uses distance measurement to identify neighboring points and can estimate missing values with the complete data of neighboring observations, was employed here to input missing values in the KIRC sample data.

### Histone data and preprocessing

We downloaded the hg19 version of the GSE86091 dataset with paracancerous samples and tumor samples from the Gene Expression Omibus (GEO) database (22). The dataset contained three histone information, namely, H3K4me1, H3K4me3, and H3K27ac.

### Identification of PCGs with epigenetic dysregulation

The R package limma (23) was used to identify differentially expressed PCGs in KIRC. The P value was determined by the Benjami-Hochberg method, and PCGs with false discovery rate (FDR)< 0.05 and |log2 fold change (FC)|> 1 were considered significant. Secondly, we screened peaks specific to KIRC based on the physical location of histones-modified peaks, and only the peaks with p< 0.05 were retained as differential peaks. Then GTF file from GENCODE was combined to obtain histone-modified differentially genes. Human enhancer database was downloaded from FANTOM5 to screen gene enhancers. A gene promoter was defined as 2 kb upstream and 0.5 kb downstream of the transcription start site (TSS). The R package ChIPseeker (24) was employed to identify gene promoter. DMR was detected using The Bumphunter method in the R package CHAMP, and the area with BumphunterDMR.p.value< 0.001 was considered as a significant DMR. Finally, PCGs abnormally regulated by epigenetics were defined by the following criteria (1): PCGs were differentially expressed in KIRC and normal samples; (2) promoters or enhancers overlapped at least one differential histone modification region or differentially methylated regions (named epi-PCG, non-epi-PCG).

### Genomic characterization of epigenetically dysregulated PCG

To compare the genomic characteristics of PCGs with or without epigenetic dysregulation, we analyzed the exons, transcripts, and number and length of the four types of genes epi-PCG and non-epi-PCG.

### PCG genomic map of epigenetic dysregulation characterized by different histone modifications

To explore the epigenetic characteristics of PCG caused by histone modification, the distribution characteristics of the promoters and enhancers of different histone modification epi-PCG on the genome were analyzed.

### Functional enrichment analysis on candidate PCGs with epigenetic dysregulation specific in KIRC

To understand the function of epigenetically dysregulated PCG, we used clusterProfiler in the R software package (v3.14.0) (25) to perform KEGG pathway analysis and GO function enrichment analysis on epi-PCGs related genes.

### Molecular subtyping of PCGs based on epigenetic dysregulation

From the TCGA and ICGC data sets, univariate analysis on epi-PCGs was performed to screen prognosis-related genes (p< 0.05), followed by molecular subtyping. Genes related to survival in the two data sets were selected as cluster genes, and the samples from the TCGA and ICGC data set were clustered by ConsensusClusterPlus (26) to determine the optimal cluster number according to the cumulative distribution function (CDF) number. Next, we compared the distribution of pathways in different subtypes, and analyzed the immune microenvironment and chances of KIRC patients benefiting from receiving immunotherapy.

### Random grouping of training set samples and single-factor analysis

A total of 526 samples in the TCGA data set were divided into a training set and a validation set. To avoid random distribution error from affecting the stability of subsequent modeling, all the samples were randomly grouped for 100 times without replacement. Here, the group sampling was performed based on the ratio of training set: verification set = 3:2. The most suitable training set and validation set was selected according to the following conditions: 1) The two groups were similar in age distribution and gender ratio; 2) The two randomly grouped data sets had similar numbers of samples after clustering the gene expression profiles. Finally, the training set and test set samples were assessed by chi-square test to validate the grouping. In the training set data, for each epi-PCG, the R package survival coxph function was used to perform univariate Cox analysis. P< 0.05 was the threshold to screen genes with prognostic significance.

### Least absolute shrinkage and selection operator (Lasso) cox regression for multi-factor risk analysis

To facilitate clinical testing, it is necessary to further reduce the number of prognostic genes in the model while maintaining a high accuracy. The Lasso method shapes a more refined model by constructing a penalty function, and it compresses certain coefficients and sets some coefficients to zero at the same time (27). This method has the advantages of subset shrinkage, and as a biased estimation for processing data with multicollinearity, it can realize the selection of variables while estimating the parameters in solving the problem of multicollinearity in regression analysis. We used glmnet in the R software package to perform lasso cox regression analysis, observed the change trajectory of each independent variable, and used 10-fold cross-validation to build the model, and analyzed the confidence interval under each lambda. Stepwise Akaike information criterion (stepAIC) (28) was employed in ensuring the statistical fit of the model and number of parameters used to fit the model. The stepAIC method in the MASS package starts with the most complex model and deletes a variable in turn to reduce the AIC, with a lower value indicating a better model. This algorithm was used here to reduce the number of genes. The RiskScore calculation formula was:


$$RiskScore=\overset{k=1}{\underset{n}{\sum^{}}}Exp_{k}*Coef_{k}$$

(Coef: regression coefficient of genes in multivariate Cox regression analysis, n: total number of genes related to prognosis). The RiskScore of each patient was calculated by the formula. Survminer R package (http://www.sthda.com/english/rpkgs/survminer/) was used to determine the optimal cut-off values. We performed z-score transformation on RiskScore, and z-score = 0 was set as a cut-off for dividing samples into high-risk groups (z-score > 0) and low-risk groups (z-score< 0). The Kaplan-Meier method was used to estimate the survival rate and survival time of different groups.

### Functional analysis on the model pathways

The R software package GSVA (29) was used to perform single-sample GSEA analysis on the gene expression profile of the samples. The score of each sample on different functions was calculated to obtain the ssGSEA score of each function in each sample, and we further determined the correlation of these functions with RiskScore.

### Cell culture

The HK2 cell line (normal human renal tubular epithelial cell line) and all the four human RCC cell lines (786-O, A498, Caki-1 and ACHN) were obtained from the Cell Bank of Type Culture Collection of the Chinese Academy of Sciences (CBTCCCAS, Shanghai, China). The cells were cultured in RPMI 1640 medium (Gibco, United States) or DMEM medium (Gibco, United States) containing 10% fetal bovine serum (Gibco, United States), 100 U/ml penicillin, and 100 mg/ml streptomycin at 37°C in a humidified incubator with 5% CO_2_.

### Quantitative real-time PCR

Total RNA extract was prepared from HK2 cells and RCC cells using TRIzol Reagent (Beijing Solarbio Technology Co., Ltd., Beijing, China) according to the manufacturer’s instructions. The reverse transcription was performed using the TaKaRa PrimeScriptTM RT-PCR kit (TaKaRa, Mountain View, CA). The qRT-PCR was conducted using the SYBR Premix Ex TaqTM (TaKaRa). Eight epi-PCGs mRNA expression levels were evaluated by the 2-ΔΔCT method. The expression of GAPDH served as an internal control. The primer sequences utilized in the present study are listed in **Supplementary Table 1**.

### Western blot

To measure the protein concentrations, RIPA lysis buffer (R0010, Solarbio, China) supplemented with protease inhibitors (Roche) was used to lyse the HK2 cell line and all four human RCC cell lines. The BCA kit (Pierce, Rockford, IL) was used to measure the protein concentrations. After adding the total protein to loading buffer, it was separated using 10% sodium dodecyl sulfate polyacrylamide gel electrophoresis (SDS-PAGE) and then transferred onto polyvinylidene fluoride (PVDF) membrane (Merck Millipore, Billerica, MA). The membrane was blocked with 5% skimmed milk for one hour and subsequently blocked with primary antibodies against ETV4 (Santa Cruz), SH2B3 (Thermo Fisher Scientific), FATE1 (Santa Cruz), GRK5 (Abcam), MALL (Santa Cruz), HRH2 (ABclonal), SEMA3G (Abcam) and SLC10A6 (Santa Cruz) overnight at 4°C. After the PVDF membrane was washed with TBST, it was incubated with the corresponding secondary antibody for two hours. Identification of the proteins was conducted using Pierce SuperSignal West Pico Chemiluminescent Substrate (Termo Fisher, Waltham, MA), following the instruction of the manufacturer. GAPDH antibody was used as an internal reference.

### Statistical analysis

R software (v4.1.0) was used to perform all statistical analysis. Student’s t test was conducted between two groups. ANOVA test was performed among three groups. Log-rank test was performed in Kaplan-Meier survival analysis, univariate and multivariate Cox regression analysis. In the relation between RiskScore and clinical features, Wilcoxon test was conducted between two groups, and Kruskal-Wallis test was conducted among four groups. Benjamini & Hochberg correction was used to adjust P values. All parameters without special indication in the methods were set as default. P< 0.05 or FDR< 0.05 was considered as significant. *P< 0.05, **P< 0.01, ***P< 0.001, ****P< 0.0001. ns, no significant.

## Results

### Identification of PCGs with epigenetic dysregulation

To analyze the relationship between PCGs expression and epigenetic changes in KIRC, limma was used to identify significantly differentially expressed genes (a total of 2755 PCGs). Combining histone modification data and methylation data, we finally found 872 epi-PCGs and 18629 non-epi-PCGs. Epi-PCGs accounted for only 4.47% of all the PCGs (872/19501).

**Figure 2:**
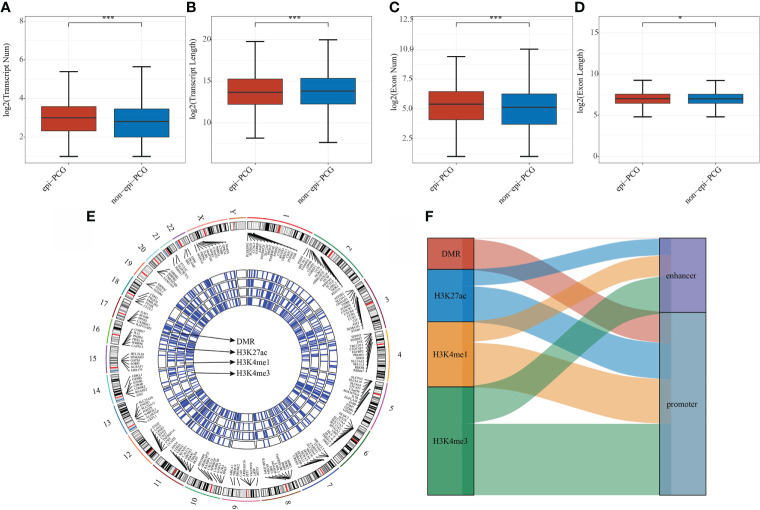
Comparison of genomic characteristics of epigenetically dysregulated lncRNA/PCGs (n = 872) and non-epigenetically dysregulated lncRNA/PCGs (n = 18629). **(A)** Comparison of the number of transcripts; **(B)** Comparison of the length of transcripts; **(C)** Comparison of the number of exons; **(D)** Comparison of the lengths of exons; **(E)** Genomic landscape of epi-PCGs characterized by histone modification; **(F)** Location distribution of histone modifications of epi-PCGs. *P < 0.05, ***P < 0.001.

The number and length of gene exons and transcripts of epi-PCGs and non-epi-PCGs was compared to show the genomic characteristics of epigenetically dysregulated PCGs. The number of epi-PCG transcripts was more than that of non-epi-PCGs, while the length of epi-PCGs transcripts was shorter than that of non-epi-PCGs (**Figures 2A, B**). Meanwhile, epi-PCGs had more exons and longer length of exons than those of non-epi-PCGs (**Figures 2C, D**). Furthermore, we systematically analyzed the epi-PCGs in KIRC, and revealed the epi-PCG landscape characterized by different histone modifications and differentially methylated regions (**Figure 1E**). The data demonstrated that most of the epigenetically dysregulated PCGs were accompanied by a variety of histone modification abnormalities, and that these abnormal histone modifications were mainly concentrated in the promoter region (**Figure 2F**).

### SsGSEA analysis of dysregulated epi-PCGs

**Figure 3:**
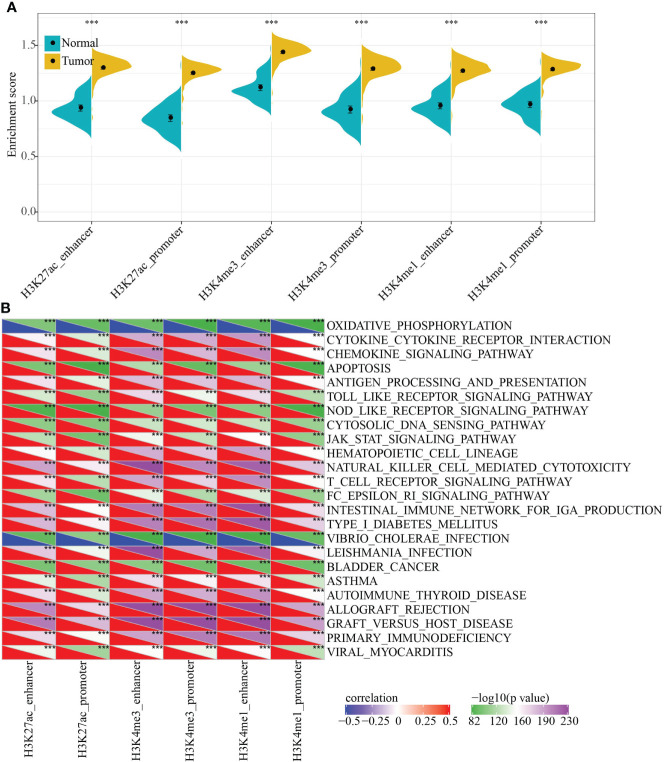
Functional enrichment analysis of epi-PCGs. **(A)** Differential expression of 6 kinds of epigenetically dysregulated PCGs in cancer (n = 319) and adjacent cancer (n = 160) tissues; **(B)** The most relevant KEGG Pathway enriched by the 6 kinds of dysregulated PCGs. ***P < 0.001.

To characterize the potential functions of PCG dysregulation caused by abnormal histone modifications, we systematically analyzed the relationship between the expression of epi-PCGs and the pathways in KIRC. Specifically, we extracted the expression profiles of PCGs caused by different histone modifications, and calculated the enrichment scores of each sample in these PCGs using ssGSEA. It was found that the GSEA scores of 6 kinds of dysregulated histones were significantly higher in tumor samples than normal samples, indicating that these dysregulated histones had cancer-promoting effect (**Figure 3A**).

In addition, we also evaluated the KEGG Pathway score of each sample and analyzed the relationship between the enrichment score of each type of epi-PCG and KEGG Pathway to obtain relevant KEGG Pathway for each type of epi-PCG. A total of 24 pathways, which were the most relevant KEGG Pathways related to the 6 types of epi-PCG, were shown in **Figure 3B**. The results indicated that different types of epi-PCG-related pathways had certain consistency. Among these 24 pathways, there were tumor-related pathways such as BLADDER_CANCER, hematopoietic cell lineage, JAK-STAT signaling pathway, immune-related pathways such as Toll like receptor signaling pathway, T cell receptor signaling pathway, natural killer cell mediated cytotoxicity. These data suggested that epi-PCGs were closely related to tumor occurrence, development and immunity.

### Epigenetic dysregulation of PCGs was closely related to RNA modification

**Figure 4:**
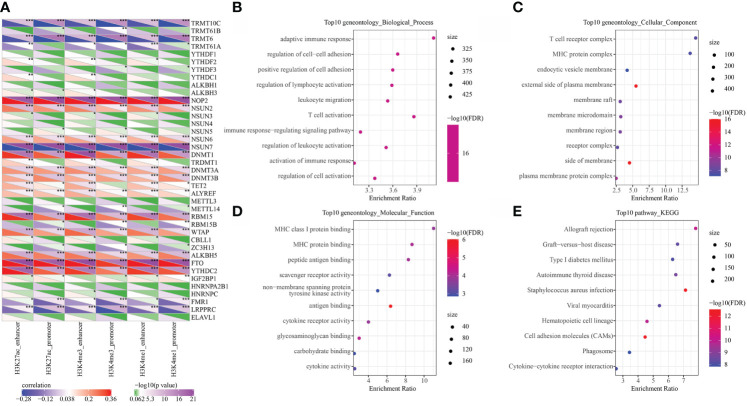
Epi-PCGs and RNA modification correlation and functional enrichment analysis. **(A)** Correlation between the enrichment scores of 6 kinds of epigenetic modification; **(B)** Epi-PCGs-enriched BP annotation map; **(C)** Epi-PCGs-enriched CC annotation map; **(D)** Epi-PCGs-enriched MF annotation map; **(E)** Epi-PCGs-enriched KEGG annotation map. The abscissa represents the enrichment score, and the ordinate represents the enriched functions or pathways. The size represents the number of gene enrichment, and the color represents P-value. *P < 0.05, **P < 0.01, ***P < 0.001.

RNA modification is an important epigenetic feature related to a variety of important biological processes. Here, we analyzed the relationship between 6 types of epi-PCGs and m6A and m5C RNA modifications. Specifically, we extracted the expression profile of m6A, m5C, and m1A in KIRC from TCGA, and the correlation between the enrichment scores of 6 types of epi-PCGs and m6A, m5C, and m1A was analyzed (**Figure 4A**). We found that these enrichment scores were significantly correlated with m6A, m5C, and m1A. The R software package clusterProfiler (v3.14.0) was further used to perform KEGG pathway analysis and GO function enrichment analysis on the epi-PCGs. For the GO function annotation of genes, 519 BPs with significant differences (FDR<0.05) were annotated; 79 CCs with significant differences (FDR<0.05) were annotated; 48 MFs with significant differences were annotated (FDR<0.05); KEGG pathway enrichment analysis were annotated to 38 significant pathways (FDR<0.05). The top 10 enriched terms were visualized (**Figures 4B** **–E**).

### Identification of 3 molecular subtypes with prognostic differences based on epi-PCGs

**Figure 5:**
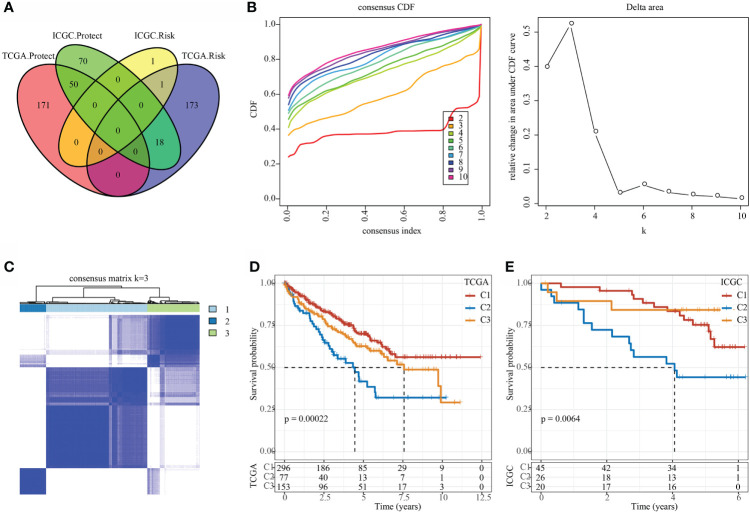
Identification of epi-PCGs-related molecular subtypes. **(A)** Venn diagram of prognostic significant genes in TCGA and ICGC data set obtained by univariate Cox regression analysis; **(B)** CDF curve and CDF Delta area curve of TCGA cohort samples (n = 526). Delta area curve of consensus clustering, which indicates the relative change in area under the cumulative distribution function (CDF) curve for each category number k compared with k – 1. The horizontal axis represents the category number k and the vertical axis represents the relative change in area under CDF curve; **(C)** Cluster heat map of TCGA data set samples (n = 526) when k = 3; **(D)** KM curve of the prognosis of the three molecular subtypes in the TCGA data set (n-C1 = 296, n-C2 = 77, and n-C3 = 153); **(E)** KM curve of the prognosis of the three molecular subtypes in the ICGC data set (n-C1 = 45, n-C2 = 26, and n-C3 = 20).

In the TCGA and ICGC data sets, single-factor survival analysis was performed on epi-PCGs, and survival-related genes in both data sets were selected as cluster genes for molecular subtyping. Finally, 51 intersection genes were included (**Figure 5A**). Analysis of expression differences of the 51 genes between normal and tumor samples showed that these genes had significant differences in expression (**Supplementary Figure 1A**). In addition, a modification map of some genes on histones was drawn (**Supplementary Figures 1B, C**). Based on 51 Epi-PCGs, the two data sets were clustered by ConsensusClusterPlus, and the optimal number of clusters was determined according to the cumulative distribution function (CDF). Combining CDF Delta area curve and survival Curve, k = 3 was used to obtain three Epi-PCGs-related subtypes (**Figures 5B, C**). KM analysis indicated that C2 had a poor prognosis in the TCGA data set, while C1 had a better prognosis (**Figure 5D**). Similar results were observed in the ICGC data set (**Figure 5E**).

**Figure 6:**
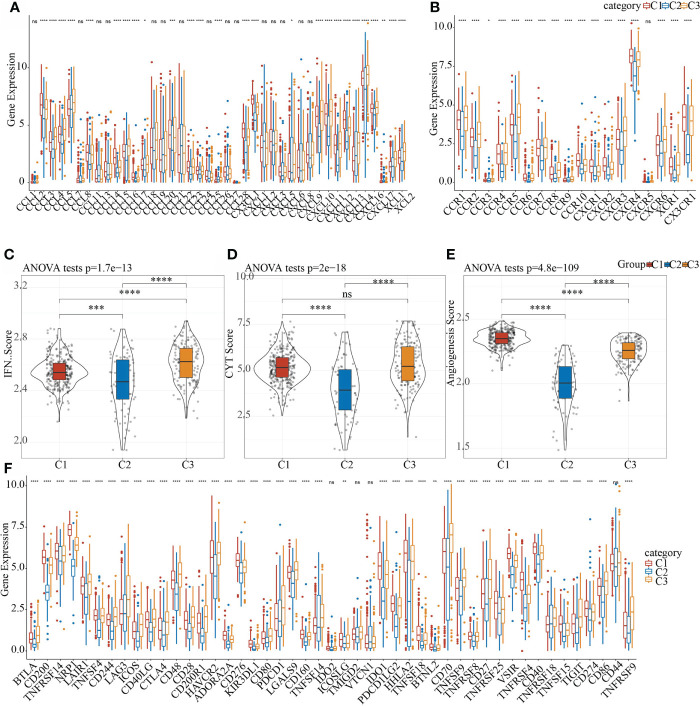
Differences in the distribution of chemokines, IFNγ scores, immune T cell cytolysis activity, angiogenesis scores, and immune checkpoint genes in different subgroups. **(A)** Difference in the expression and distribution of chemokines in the TCGA cohort; **(B)** Difference in the expression and distribution of chemokine receptors in the TCGA cohort; **(C)** Difference in the distribution of IFNγ scores in different subgroups in the TCGA cohort; **(D)** Differences in immune T cell cytolysis activity in different subgroups; **(E)** Differences in angiogenesis scores in different subgroups; **(F)** Differences in the expression and distribution of immune checkpoint genes in the TCGA cohort; the significance was tested by analysis of variance, * means p< 0.05; ** means p< 0.01, *** means p< 0.001, **** means p< 0.0001, ns, not significant.

Studies found that chemokines play a key role in the occurrence and development of tumors. They can mediate a variety of immune cells into the tumor microenvironment, help T cells enter tumor and affect tumor immunity and therapeutic effects. Therefore, we analyzed whether there were expression differences in chemokines among the three molecular subtypes. In the TCGA data set, 26 of 41 chemokines (63.41%) showed significant expression differences in different subtypes (**Figure 6A**), which suggested that the degree of immune cell infiltration of different subtypes may be different, and that these differences could lead to differences in tumor progression and immunotherapy effects. In addition, 17 of the 18 chemokine receptor genes (94.44%) had significant differences in the expression of the three molecular subtypes (**Figure 6B**).

CD8 + T cells in the tumor microenvironment can produce interferon-γ (IFNγ) to stimulate the up-regulation of PD-1/PD-L1 and IDO1 gene expression (30, 31). Studies have shown that the up-regulation of IDO1 expression is positively correlated with poor prognosis and tumor progression and metastasis (32, 33). We extracted Th1/IFNγ gene signatures and 47 immune checkpoint-related genes from a previous study (34). In addition, according to Rooney Michael S (35), the average value of GZMA and PRF1 expression levels was used to evaluate the immune cytolysis (CYT) of immune t cell of each patient, then the angiogenesis-related gene set was obtained to evaluate each patient’s angiogenesis score (36). The IFNγ score, CYT score and angiogenesis score of each patient were calculated using ssGSEA. It can be observed that there were significant differences in IFNγ scores in each subgroup (**Figure 6C**). Among them, C1 and C3 had the highest immune T cell cytolysis activity, while that of C2 was the lowest (**Figure 6D**). C2 had the lowest angiogenesis score (**Figure 6E**). In the correlation analysis of 47 immune checkpoint-related genes, 43 genes had significant differences in the three subgroups (**Figure 6F**). These results indicated that different subgroups may respond to immunotherapy differently.

### The immune characteristics and pathway characteristics of different molecular subtypes were significant

**Figure 7:**
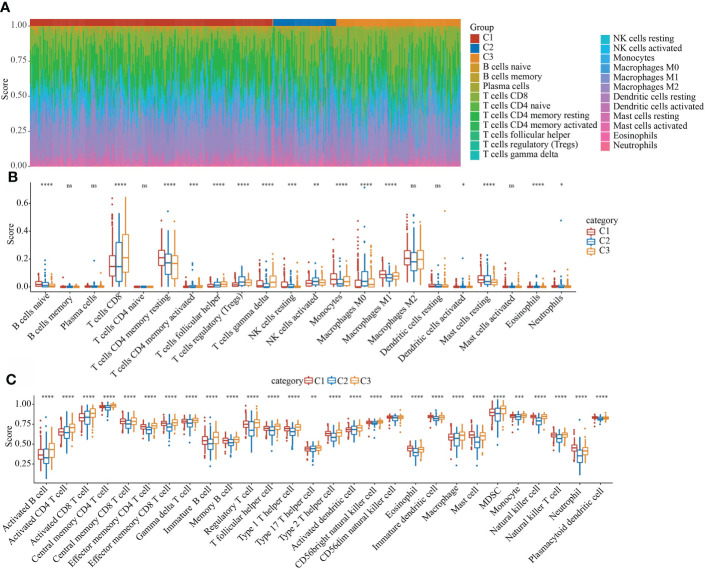
Evaluation of immune and pathway characteristics of different molecular subtypes. **(A)** The proportion of the 22 immune cell components of samples in different subgroups. **(B)** Differences in 22 immune cell components of samples in different subgroups; **(C)** Differences in 28 immune infiltration scores in different subgroups. *P < 0.05, **P < 0.01, ***P < 0.001. ns, not significant.

In the TCGA data set, the CIBERSORT method was used to evaluate the scores of 22 immune cells in each sample, and the distribution of these immune cell scores in the three subgroups was observed, as shown in **Figure 7A**. 16 immune cells showed significant differences in different subtypes (**Figure 7B**). We used the method of ssGSEA to calculate the scores of 28 immune cells (37), then compared their differences in the subtypes, and 28 immune infiltration scores were found to have significant differences in the subtypes (**Figure 7C**).

### C2 molecular subtype had a lower TIDE score

We analyzed the differences of different molecular subtypes in response to immunotherapy and chemotherapy. TIDE (http://tide.dfci.harvard.edu/) software was used to evaluate the potential clinical effects of immunotherapy on our defined molecular subtypes. A higher TIDE prediction score indicated a higher possibility of immune escape, which suggests that the patient is less likely to benefit from immunotherapy. As shown in **Supplementary Figure 2**, in the TCGA data set, C2 had the lowest TIDE score (**Supplementary Figure 2A**). At the same time, we also compared the predicted T cell dysfunction scores (**Supplementary Figure 2B**) and T cell exclusion scores (**Supplementary Figure 2C**) in different molecular subtypes, and there were also significant differences between different groups.

### Establishing a prognostic risk model based on epi-PCG-related genes

The final training set data had a total of 316 samples, and the test set data had a total of 210 samples. See **Table 2** for sample information of training set and validation set. Chi-square test was applied to assess the training set and test set samples. The results showed that our grouping was reasonable and there was no significant difference between groups (P > 0.05).

**Table 2:** Clinical information of TCGA training set and validation set samples.

Clinical Features	TCGA-Train	TCGA-test	P-Value
Gender			
Male	206	137	1
Female	110	73	
T Stage			
T1	158	109	0.6742
T2	46	23	
T3	106	73	
T4	6	5	
N Stage			
N0	141	97	0.3379
N1	7	9	
NX	168	104	
M Stage			
M0	275	161	0.0786
M1	37	43	
MX	4	6	
Stage			
I	157	104	0.1018
II	39	18	
III	81	42	
IV	39	43	
X	0	3	
Grade			
G1	11	2	0.0911
G2	132	94	
G3	127	78	
G4	39	35	
GX	7	1	
Age			
>60	163	99	0.3637
<=60	153	111	

### Construction and evaluation of an 8-gene risk model

**Figure 8:**
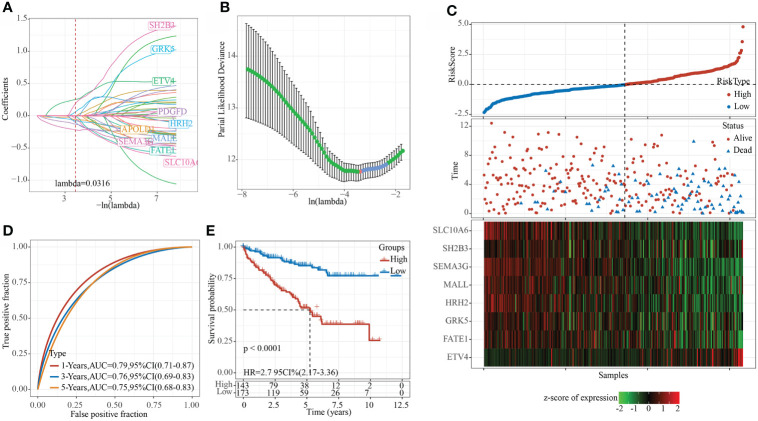
Constructing a prognostic model in TCGA data set. **(A)** The change trajectory of each independent variable, the horizontal axis represents the log value of the independent variable lambda, and the vertical axis represents the coefficient of the independent variable; **(B)** The confidence interval under each lambda. **(C)** RiskScore, survival time and survival status and expression of the 8 genes in the TCGA training set; **(D)** ROC curve and AUC of 8-gene signature classification; **(E)** KM survival curve distribution of the 8-gene signature in the training set.

Using the training set data, univariate Cox analysis was performed for each epi-PCG, and p< 0.05 was the threshold for filtering. Finally, 46 prognostic genes were included. In this study, 46 genes with differences have been identified. We used the R software package glmnet to perform lasso cox regression analysis. Firstly, the change trajectory of each independent variable was analyzed, as shown in **Figure 8A**. It can be seen that as the lambda gradually increased, the number of independent variable coefficients close to 0 also gradually increased. 10-fold cross-validation was employed to build a model, and the confidence interval under each lambda was determined, as shown in **Figure 8B**. It can be seen that the model was optimal when lambda = 0.0316. Thus, 10 genes when lambda = 0.0316 were considered as the target genes for further analysis. To reduce the number of genes, the stepAIC method in the MASS package was used, we finally reduced 10 genes to 8 genes. The final RiskScore formula was as follows:

RiskScore=0.28*ETV4+0.631*SH2B3-0.338*FATE1+0.363*GRK5-0.42*MALL-0.196*HRH2-0.354*SEMA3G-0.431*SLC10A6

The RiskScore of each sample was calculated according to the expression level of the samples, and the RiskScore of the sample was shown in **Figure 8C**. Furthermore, we used the R software package timeROC to analyze the ROC of RiskScore for prognostic classification, and determined the classification efficiency of 1-, 3-, and 5- year prognosis, respectively. As shown in **Figure 8D**, the model had a high AUC area. Finally, z-score was performed on Riskscore. Samples with Riskscore greater than zero were divided into high-risk groups, while those with Riskscore lower than zero were in low-risk groups, and the KM curve was drawn, as shown in **Figure 8E**. A significant difference of p< 0.0001 can be found, and here 143 samples were classified into high-risk groups and 173 samples were classified into low-risk groups.

### The 8-gene signature had a strong robustness in different cohorts

To evaluate the robustness of the model, the RiskScore of each sample in the TCGA validation set, TCGA entire data set and ICGC data sets were calculating using the same model and the same coefficients as the training set, according to the expression level of the sample. The R software package timeROC was applied to analyze the prognostic classification of the RiskScore of the TCGA validation set. The ROC efficiencies of 1, 3, and 5 years were 0.73, 0.69, and 0.63, respectively (**Supplementary Figure 3A**). Finally zscore was performed on the Riskscore. Samples with Riskscore greater than zero were divided into high-risk group, whereas those lower than zero were in low-risk group, and the KM curve was drawn. The results showed that the prognosis of patients in the high-risk group was significantly worse than that of the low-risk group (p< 0.05, **Supplementary Figure 3B**). Specifically, 99 samples were classified as high-risk, and 111 samples were classified as low-risk.

In all TCGA data sets, the ROC efficiencies of 1, 3, and 5 years were 0.77, 0.73, and 0.70, respectively (**Supplementary Figure 3C**). The prognosis of patients in the high-risk group was significantly worse than that of the low-risk group (p< 0.001, **Supplement Figure 3D**). Here, 242 samples were classified as high-risk group, and 284 samples were classified as low-risk group.

Furthermore, we used the independent verification set ICGC to verify the applicability of the model. TimeROC was employed to assess the prognostic classification of the RiskScore on ICGC. The ROC efficiencies of 1, 3, and 5 years were 0.77, 0.73, and 0.70, respectively (**Supplementary Figure 3E**). Z-score on Riskscore was then performed, and samples with a Riskscore greater than zero were divided into the high-risk group, while those lower than zero were in the low-risk group, and the KM curve was drawn. The results demonstrated that the prognosis of patients in the high-risk group was significantly worse than that of the low-risk group (p< 0.001, **Supplementary Figure 3F**). Of these, 49 samples were classified as high-risk group, and 42 samples were classified as low-risk group.

### Riskscore can distinguish different clinical subgroup characteristics

**Figure 9:**
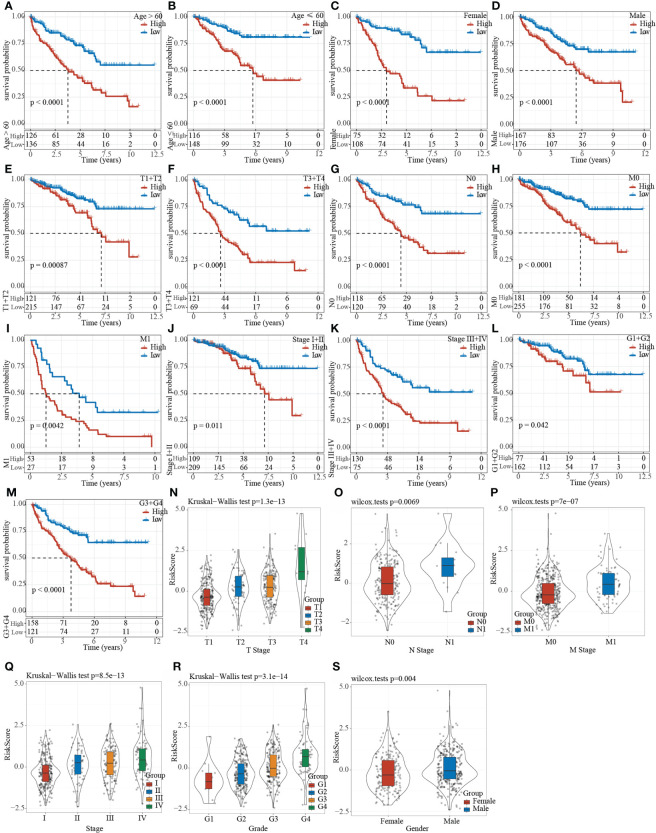
Clinical subgroup survival analysis and correlation analysis based on Riskscore. **(A–M)** Prognostic survival curve of clinical characteristics based on Riskscore; **(N–S)** RiskScore comparison in clinical characteristics of TCGA data set.

The clinical subgroup characteristics were divided by the Riskscore into high- and low-risk groups. The results demonstrated that Riskscore can significantly distinguish Age, Gender, TMN stage and Grade into two groups with prognostic differences (**Figures 9A** **–M)**. Furthermore, comparison on the correlation between RiskScore and clinical subgroup characteristics also showed significant differences of Riskscore in T Stage, N Stage, M Stage, Stage, Grade, and Gender (**Figures 9N** **–S**, p< 0.05).

### The relationship between RiskScore and the pathways

We calculated the ssGSEA score of each sample on different functions, and further analyzed the correlation between these functions and RiskScore. The functions with a correlation greater than 0.4 were selected and shown in **Supplementary Figure 4A**, from which it could be found that one function was positively correlated with the RiskScore, whereas the remaining 22 were negatively correlated with the RiskScore. The most relevant 23 KEGG Pathways were selected and subjected to cluster analysis based on their enrichment scores, as shown in **Supplementary Figure 4B**. Among the 23 pathways, for example, P53 signaling pathway, increased with the increase of RiskScore, while metabolic pathways such as fatty acid metabolism, glycolysis gluconeogenesis gluconeogenesis, galactose metabolism decreased with the increase of RiskScore. Moreover, we characterized the protein-protein interaction (PPI) among these eight prognostic genes using the STRING online tool (https://www.networkanalyst.ca/). The result showed that SEMA3G, ETV4, and SH2B3 had a close interaction, and that GRK5 and HRH2 had a close interaction (**Supplementary Figure 5**), suggesting that they may have a synergetic effect on affecting KIRC prognosis.

### The expression of the eight prognostic genes was correlated with immune infiltration and was differential in the three molecular subtypes

Furthermore, we evaluated whether there was a correlation between the expression of prognostic genes and immune cell infiltration. Pearson correlation analysis revealed that the enrichment of M0, M1, and M2 macrophages, and regulatory T cells was obviously correlated with the prognostic genes (**Supplementary Figure 6**). Especially, a relatively strong correlation was observed between ETV4 and activated CD4 memory T cells (R = 0.30). SEMA3G, SLC10A6, and SH2B3 expression were significantly correlated with regulatory T cells (R = -0.32, -0.38, and -0.35, respectively). In addition, we found the distribution of the expression of eight prognostic genes in three molecular subtypes. C2 subtype with the worst overall survival had the lowest expression of all eight genes in both TCGA and ICGC datasets (**Supplementary Figure 7**), which was consistent with the previous result that high-risk group had relatively lower expression of these genes (**Figure 8C**).

### The 8-gene signature was an independent prognostic risk factor for KIRC

To validate the independence of the 8-gene signature model in clinical applications, single-factor and multi-factor cox analysis were performed on the TCGA data set. Univariate COX regression analysis demonstrated that RiskType was significantly related to patients’ survival. Corresponding multivariate COX regression analysis showed that RiskType (HR = 1.77, 95%CI = 1.39–2.24, p< 1e-5) was still closely related to survival. The above results indicated that the 8-gene signature was an independent prognostic risk factor for KIRC (**Supplementary Figures 8A, B**).

A nomogram is more effective to display the results of the risk model, and it is more convenient to be applied for predicting the outcome. The nomogram uses the length of the straight line to indicate the degree of influence of different variables on the outcome and the influence of different values of the variables on the outcome. We combined the significant clinical features of Age, M Stage, and RiskScore in multi-factor cox analysis to construct a nomogram model (**Supplementary Figure 8C**). The results demonstrated that RiskScore feature had the greatest impact on the survival rate prediction, indicating that the risk model established based on 5 genes can better predict the prognosis. In addition, we corrected the nomogram data for 1-, 3-, and 5-year survival to visualize the performance of the nomogram (**Supplementary Figure 8D**), proving that the method had a strong prediction performance. Furthermore, we plotted the DCA diagrams of Age, M Stage, RiskScore and nomogram, and the results showed that our nomogram had a high net benefit (**Supplementary Figure 8E**).

### Comparison of risk models with other models

**Figure 10:**
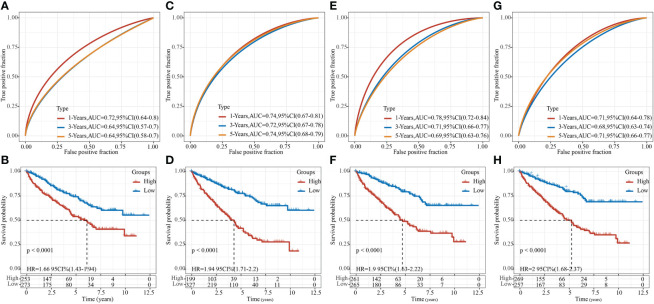
Comparison of our risk model with other models. **(A)** ROC of 9-gene signature (Zhong) risk model. **(B)** KM curve of 9-gene signature (Zhong) risk model on high- (n = 253) and low-group (n = 273) samples; **(C)** ROC of 7-gene signature (Jiang) risk model. **(D)** KM curve of 7-gene signature (Jiang) risk model on high- (n = 199) and low-group (n = 327) samples; **(E)** ROC of 7-gene signature (Chen) risk model. **(F)** KM Curve of 7-gene signature (Chen) risk model on high- (n = 261) and low-group (n = 265) samples; **(G)** ROC of the 6-gene signature (Ren) risk model. **(H)** KM curve of 6-gene signature (Ren) risk model on high- (n = 269) and low-group (n = 257) samples.

After referring to the literature, we finally selected 4 prognostic-related risk models, namely, 9-gene signature (Zhong) (38), 7-gene signature (Jiang) (39), 7-gene signature (Chen) (40), and 6-gene signature (Ren) (41), for comparing the prediction performance with our 8-gene model. To make the model comparable, we calculated the riskscore of each KIRC sample in the TCGA using the same method based on the corresponding genes in the 4 models. Z-score was performed on RiskScore, and samples with RiskScore greater than zero were classified into the high-risk group, while those with RiskScore lower than zero were in the low-risk group. The prognostic difference of KIRC samples between the two groups was calculated. The ROC and KIRC-KM curves of the four models were shown in **Figure 10**. It can be seen that the 1, 3, and 5-year AUC values of the 9-gene signature (Zhong) model were all lower than our model (**Figure 10A**); the 1- and 3-year AUC values of the 7-gene signature (Jiang) (**Figure 10C**) and 6-gene signature (Ren) (**Figure 10G**) models were lower than our model, but the 5-year AUC value was higher than our model; the 1-year AUC value of the 7-gene signature (Chen) model was higher than our model, but the 3- and 5-year AUC values were lower than our model (**Figure 10E**). The KIRC prognosis of the high- and low group samples predicted by these five models were also different (log rank p< 0.05) (**Figures 10B, D, F, H**).

### Verification of the expression level of 8 epi-PCGs *in vitro*

**Figure 11:**
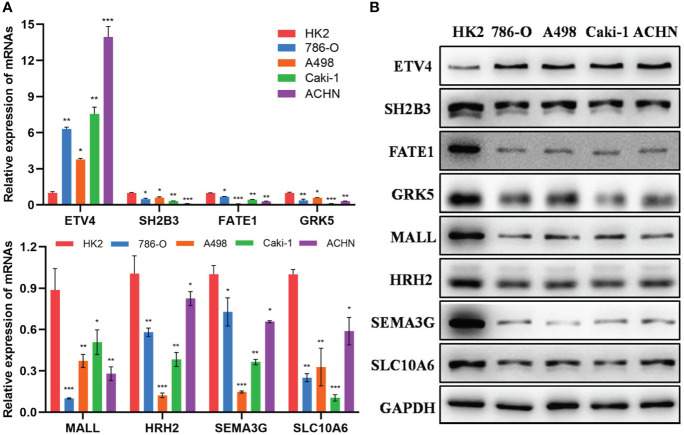
Verification of the expression level of 8 epi-PCGs *in vitro*. **(A)** The mRNA expression level of 8 epi-PCGs (ETV4, SH2B3, FATE1, GRK5, MALL, HRH2, SEMA3G and SLC10A6) in 4 kidney cancer cell lines and HK2 cell line. **(B)** The protein expression level of 8 epi-PCGs. *P < 0.05, **P < 0.01, ***P < 0.001.

Furthermore, we detected the mRNA and protein expression levels of 8 epi-PCGs (ETV4, SH2B3, FATE1, GRK5, MALL, HRH2, SEMA3G and SLC10A6) in 4 human kidney cancer cell lines (786-O, A498, Caki-1 and ACHN) and the normal human renal tubular epithelial cell line HK2. As shown in **Figure 11A**, we observed that the mRNA expression level of ETV4 was significantly increased and the expression levels of SH2B3, FATE1, GRK5, MALL, HRH2, SEMA3G and SLC10A6 were decreased prominently in kidney cancer cells when compared with HK2 cell line. The protein expression level of 8 epi-PCGs was similar to the mRNA expression level (**Figure 11B**). These findings were consistent with the bioinformatics results, indicating that the differentially expressed epi-PCGs identified in multi-omics data analysis exhibited significant changes in cancer cells.

## Discussion

Surgery is currently the main treatment for KIRC, but about 20% of KIRC patients are already at advanced stage by the time of diagnosis and have missed the optimal opportunity for taking surgery (42). Moreover, even with surgical resection, about 30% of patients with localized KIRC tend to develop recurrence and metastasis, and the 5-year survival rate of patients with distant metastasis is about 8-10% (43, 44). Therefore, there is an urgent need to further understand the molecular mechanism of KIRC occurrence and development to provide more accurate and effective clinical treatment strategies.

Dysregulation of expression of functional proteins in the cell plays a critical role in tumorigenesis, which mostly stems from the dysregulation of expression of its protein-coding genes (PCG). Based on this, we first screened differentially expressed PCGs in KIRC, and then combined with histone modification data and methylation data, we found 872 epi-PCGs and 18629 non-epi-PCGs. Epi-PCGs accounted for only 4.47% of all the PCGs. Although the proportion of epi-PCG was not high, it still pointed to the important role of epigenetic modification in tumors. Previous studies have shown that epigenetic dysfunction, including DNA methylation and histone modification, may have an important impact on the proliferation, apoptosis, migration and invasion of cancer cells. Abnormal epigenetic modifications are detected in a variety of tumor cells (45–48). Further research results showed that epi-PCGs had more transcripts and exons than non-epi-PCGs, but the transcript length was relatively short, indicating that although epi-PCGs accounted for a relatively small proportion, it is possible that the level of transcription protein was decreased. These epi-PCG-related pathways include “bladder cancer”, “hematopoietic cell lineage” (49–51), and “JAK-STAT signaling pathway” (52–54), which are related to tumor progression, indicating that these epi-PCGs play a pivotal role in the occurrence and development of tumors.

To realize clinical application of these epi-PCGs, we established a prognostic gene signature related to epi-PCGs. Lasso regression analysis demonstrated the combination with the largest frequency of occurrence that included 8 genes, namely ETS variant 4 (ETV4), SH2B adapter protein 3 (SH2B3), Fetal and adult testis-expressed transcript protein (FATE1), G protein-coupled receptor kinase 5 (GRK5), MAL-like protein (MALL), Histamine H2 receptor (HRH2), Class-3 semaphorins (SEMA3G) and Solute carrier family 10 member 6 (SLC10A6). The role of these genes in tumors has been reported, but the current research results showed that their contradictory roles in tumors. ETV4, also known as polyomavirus enhancer activator 3 protein (Pea3), is an important member of the ETS transcription factor family. Studies have shown that ETV4 is abnormally expressed in a variety of tumors, and promotes tumor progression through stimulating tumor cell proliferation and metastasis (55–57). SH2B3 is a member of the SH2B family of adaptor proteins, playing a role in negative feedback loop that controls cell growth, development and survival signals. Activated target kinase also induces SH2B3 expression and activation through phosphorylation (58). In tumors, SH2B3 usually changes its role in tumors due to mutations. In leukemias, the enrichment of SH2B3 aberrations may indicate that the loss of SH2B3 contributes to the disease progression and increases the sensitivity of leukemias to Tyrosine kinase inhibitors (59, 60). FATE1 is a gene expressed in fetal and adult testis. In normal tissues, the expression of FATE1 is mainly restricted to the testis and adrenal glands (61), and its expression is up-regulated in a variety of cancers. GRK5 affects the migration of non-small cell lung cancer cells through vinculin (62), moreover, it shows a high expression in breast cancer cells, promotes breast cancer cell metastasis, and is therefore a target for breast cancer treatment (63). MAL-like protein has a transport function, but its molecular role is largely unclear. MALL is normally expressed in laryngeal epithelial cells, and its expression changes in the early stage of carcinogenesis. The expression of MAL is significantly down-regulated (64), and it plays an important role as a binding gene of MUC1 in breast cancer (65). HRH2 is a member of the G protein-coupled receptor family widely expressed in the gastrointestinal tract, and its activity is mediated by cAMP. It has been found that the HRH2 blocker nizatidine can be used for treating advanced liver disease and liver cancer, and is a potential clinical target for liver cancer treatment (66). SEMA3 is the only group of secreted proteins in vertebrate semaphores. They are further subdivided into seven members (SEMA3A to SEMA3G). The members of the SEMA3 family have both tumor-promoting and anti-tumor functions, which are related to cell type and environment (67). SEMA3G has anti-migration and anti-invasion effects on gliomas (68), and is a prognostic gene of KIRC (69). SLC10A6 has been limitedly researched in tumors. Studies have shown that it is widely expressed in breast cancer and promotes breast cancer cell proliferation (70). These results indicated that these genes play an important role in the occurrence and development of tumors in different forms, and may also function critically in the prognosis of KIRC, but this requires further verification.

We also analyzed the RiskScore in different clinical characteristics, and found that for tumors with poor differentiation and higher malignancy (T3-4, N+, M1, and 3), the score was higher, and the prognosis of patients in the high-scoring group with different clinical characteristics was poor. Univariate and multivariate COX regression analysis results showed that RiskScore was an independent prognostic factor for KIRC. The nomogram results confirmed that RiskScore had the strongest ability in accurately predicting the prognosis of KIRC, exceeding the existing TNM and staging. For some clinical stages, the clinical application significance of the risk scoring system constructed in this study was greater. In addition, the RiskScore model was compared with the previous five assessment models. The prognosis of KIRC in the high- and low-risk samples of these five models were different, but our model had a higher AUC value in one of or some of 1-, 3- or 5- year survival predictions. This indicated that the model we developed based on differentially expressed genes combined with epigenetics can better indicate the occurrence and development of KIRC, and was a more effective model, further illustrating the clinical feasibility of our model. On the other hand, as the RiskScore changed, the pathways involved in tumor occurrence and progression were different. For example, we found that among the higher-scored pathways, the enrichment score of pathways such as P53 signaling pathway (71) increased with the increase of RiskScore, while the enrichment score of metabolic pathways such as fatty acid metabolism (72), glycolysis gluconeogenesis (73), galactose metabolism (74) decreased with the increase of RiskScore. Previous literature reports have shown that these pathways are all involved in tumor progression, but they may play different roles in different tumors, and this also requires follow-up research for verification.

Although previous research developed a series of gene signatures for kidney cancer based on epigenetic dysregulation, they focused on m6A regulators or only included limited cancer samples (17–19). Compared to the previous research, the advantage of our study was that we performed a comprehensive analysis on epigenetic dysregulation using multiple data sets. Importantly, we uncovered the relation between epigenetic dysregulation and tumor microenvironment from different aspects such as immune checkpoints, cytokines, immune cells and immune signatures. The different performance of three molecular subtypes to immune checkpoint blockade also demonstrated the important role of epigenetic dysregulation or identified epi-PCGs in tumor microenvironment modulation. The observations highlighted the potential of epi-PCGs serving as prognostic biomarkers for renal cell carcinoma. Compared with the gene signatures of KIRC in the previous studies, our 8-gene prognostic model manifested a higher AUC, which further indicated the critical role of epi-PCGs in the KIRC development and progression. Notably, we verified the expression level of the eight epi-PCGs in kidney cancer cell lines, and the results showed a consistency with the bioinformatics analysis, which further demonstrated the reliability of our analysis and the importance of the eight epi-PCGs in kidney cancer development.

However, in this study, we only analyzed the effect of differential expression of PCGs on KIRC, but did not include the abnormal expression of other non-transcriptional genes. Also, such expression difference lacked verification *in vivo* and *in vitro*. Similarly, the 8-gene signature was only preliminary screened as part of the prognostic RiskScore, but there was a lack of specific research on the role of these genes in KIRC and the detailed relationship among these genes. We will further supplement verification study *in vivo*. In addition, there are some contradictions between some research results in this study and previous research results, and have not yet been fully explained only according to our existing research results.

In conclusion, in this study, we systematically analyzed the abnormal expression of PCGs in KIRC, and combined with histone modifications, we screened 872 epi-PCGs and 18629 non-epi-PCGs. Based on the differentially expressed epi-PCGs-related genes, KIRC samples were divided into three subtypes, and these subtypes showed significant differences in prognosis. Based on the epi-PCGs, we constructed an 8-gene prognostic risk models that had a strong stability and predictive performance in both the training set and the validation set, and different RiskScores can fully reflect the clinical characteristics of patients. Compared with other existing models, our model had a higher predicting performance. The current findings help better understand the abnormal epigenetic regulation of PCG expression in KIRC. This model is expected to guide clinicians in the prognosis prediction and clinical diagnosis and treatment of KIRC patients with different phenotypes.

## Data availability statement

The datasets presented in this study can be found in online repositories. The names of the repository/repositories and accession number(s) can be found in the article/**Supplementary Material**.

## Author contributions

ZW and XL designed the study. LX, SW, RH and SL collected and analyzed data. LX and SW drafted the manuscript. ZW and XL reviewed and revised the manuscript. The manuscript has been approved by all authors for publication.

## Conflict of interest

The authors declare that the research was conducted in the absence of any commercial or financial relationships that could be construed as a potential conflict of interest.

## Publisher’s note

All claims expressed in this article are solely those of the authors and do not necessarily represent those of their affiliated organizations, or those of the publisher, the editors and the reviewers. Any product that may be evaluated in this article, or claim that may be made by its manufacturer, is not guaranteed or endorsed by the publisher.

## Supplementary material

Supplementary Figure 1 Prognosis-related epi-PCGs expression and RNA modification. **(A)** Analysis of the differential expression of prognostic-related epi-PCGs in normal samples and tumor samples. **(B–C)** Histone modification profile of epi-PCGs

Supplementary Figure 2 Comparison of TIDE score, T cell dysfunction score and T cell rejection score of molecular subtypes. **(A)** In the TCGA data set, TIDE scores are different in the three molecular subtypes. **(B)** In the TCGA data set, T cell dysfunction scores are different in the three molecular subtypes. **(C)** In the TCGA data set, T cell exclusion scores are different in the three molecular subtypes.

Supplementary Figure 3 **(A)** ROC curve and AUC of the 8-gene signature in TCGA test set; **(B)** KM survival curve of 8-gene signature in TCGA test set. **(C)** ROC curve and AUC of 8-gene signature in TCGA entire data set; **(D)** KM survival curve of 8-gene signature in TCGA entire data set. **(E)** ROC curve and AUC of 8-gene signature in the ICGC cohort; **(F)** KM survival curve of 8-gene signature in the ICGC cohort.

Supplementary Figure 4 The relationship between RiskScore and the pathways. **(A)** The correlation coefficient clustering of KEGG pathways greater than 0.4 and the RiskScore; **(B)** ssGSEA scores of KEGG pathways with a correlation greater than 0.4 in each sample as the RiskScore increases, the horizontal axis represents the sample, and the Riskscore increases from left to right.

Supplementary Figure 5 The PPI analysis for the eight prognostic genes.

Supplementary Figure 6 Pearson correlation analysis between the eight prognostic genes and the ssGSEA score of immune cells. Red and blue indicates positive and negative correlations respectively.

Supplementary Figure 7 The expression of eight prognostic genes in three molecular subtypes in TCGA **(A)** and ICGC **(B)** datasets. Kruskal-Wallis test was performed. ns, no significance. ****P < 0.0001.

Supplementary Figure 8 Single-factor and multi-factor cox analyses on the 8-gene signature**(A)** Single-factor analysis of TCGA entire data set; **(B)** Multi-factor analysis of TCGA entire data set. **(C)** Nomogram constructed with clinical characteristics and RiskScore; **(D)** Correction chart of survival rate of nomogram; **(E)** DCA chart.
