# Supplementary figures and images for: Identification of epigenetic dysregulation gene markers and immune landscape in kidney renal clear cell carcinoma by comprehensive genomic analysis
Source: Front Immunol. 2022 Aug 18;13:901662. doi: 10.3389/fimmu.2022.901662 (PMC9433776; doi:10.3389/fimmu.2022.901662)

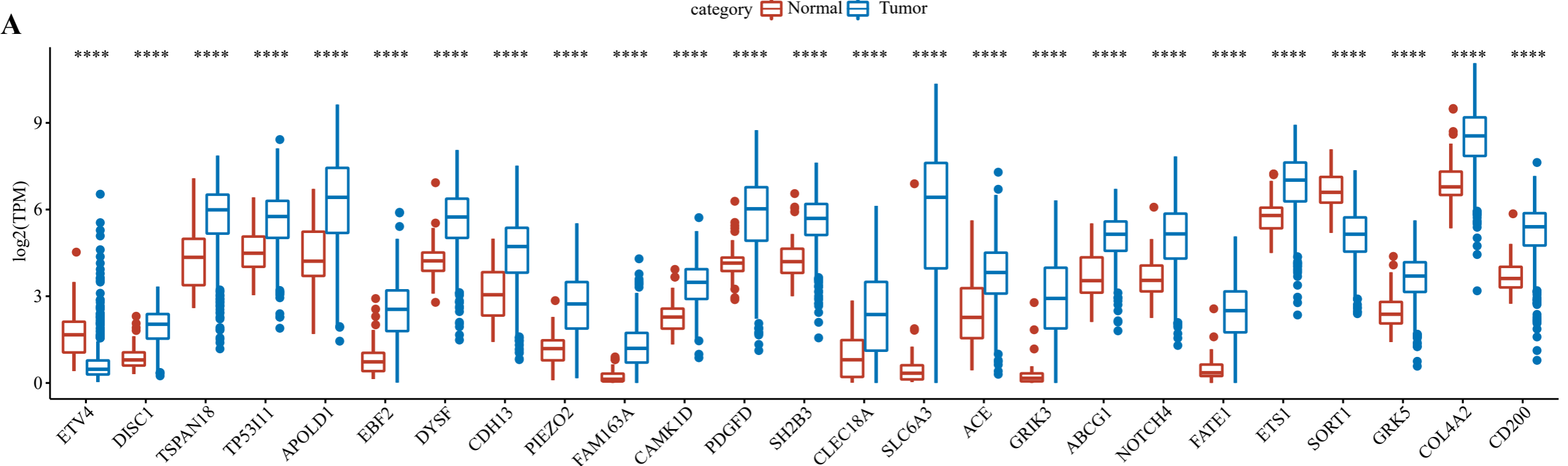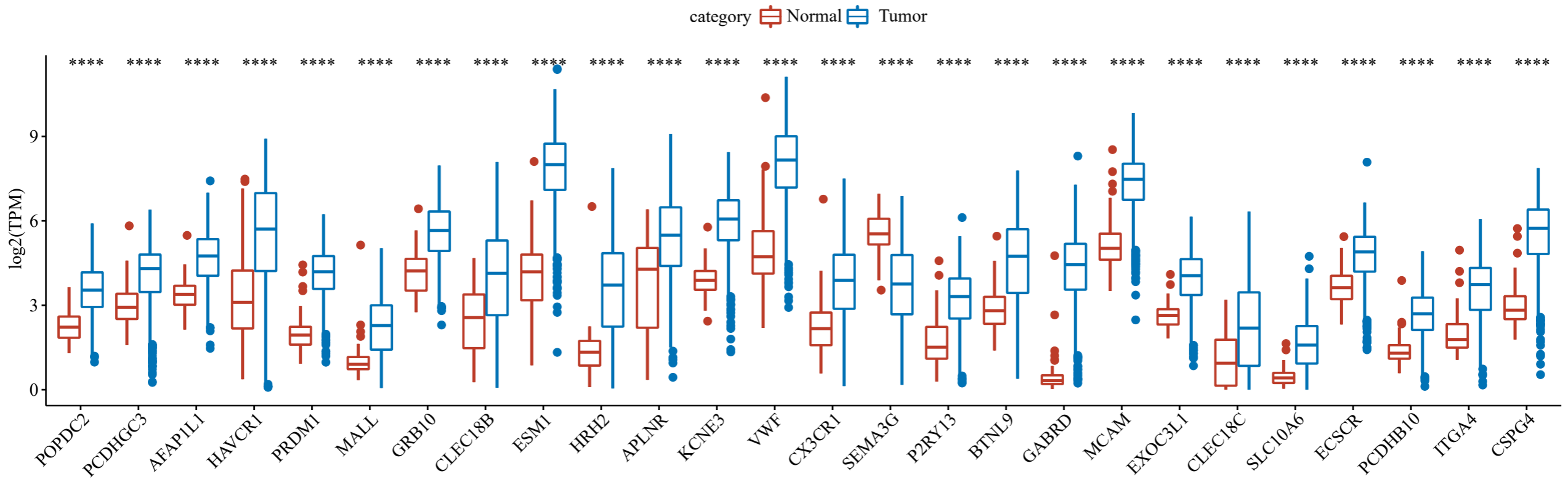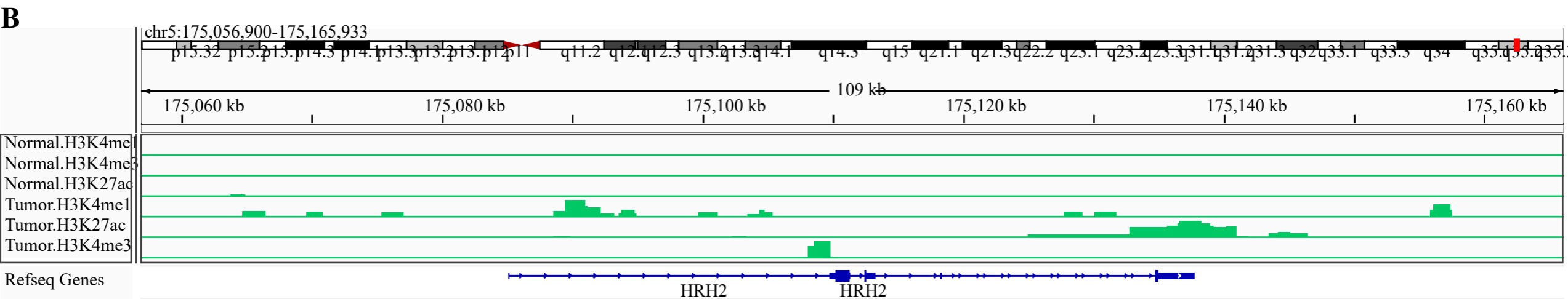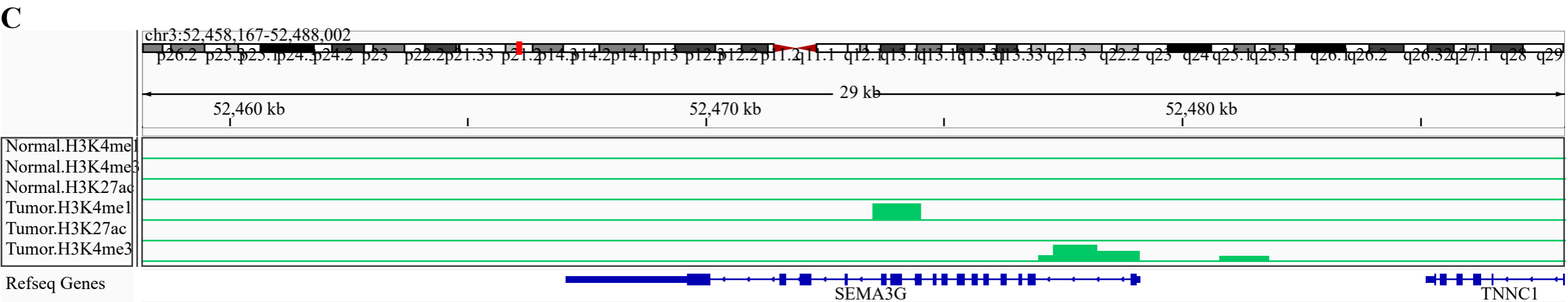

Supplement: Supplementary Figure 1 — Prognosis-related epi-PCGs expression and RNA modification. (A) Analysis of the differential expression of prognostic-related epi-PCGs in normal samples and tumor samples. (B–C) Histone modification profile of epi-PCGs [file DataSheet_1.pdf]

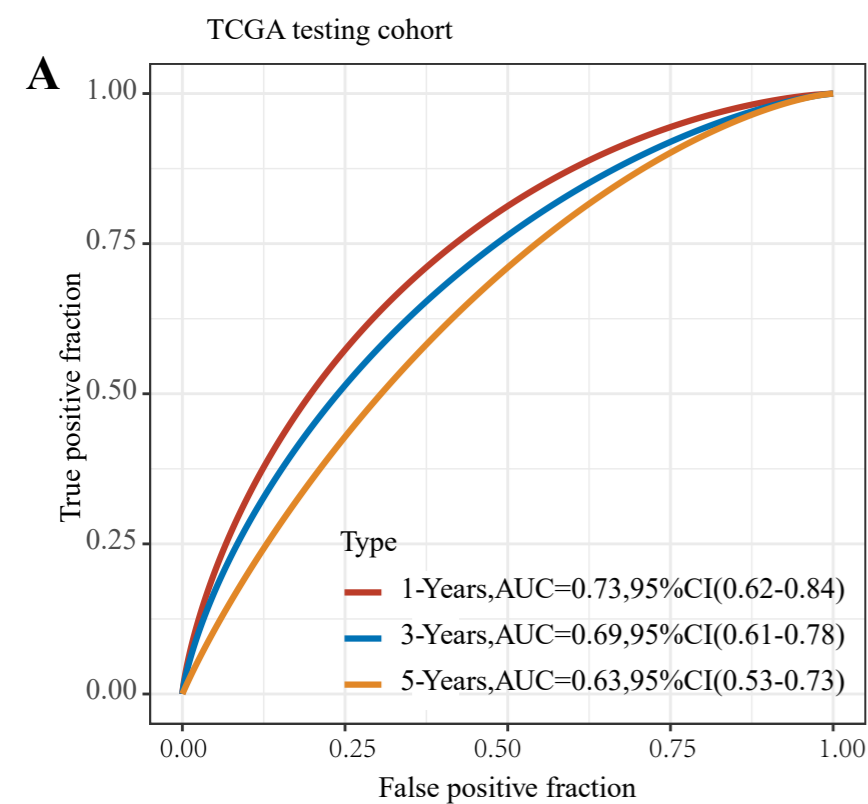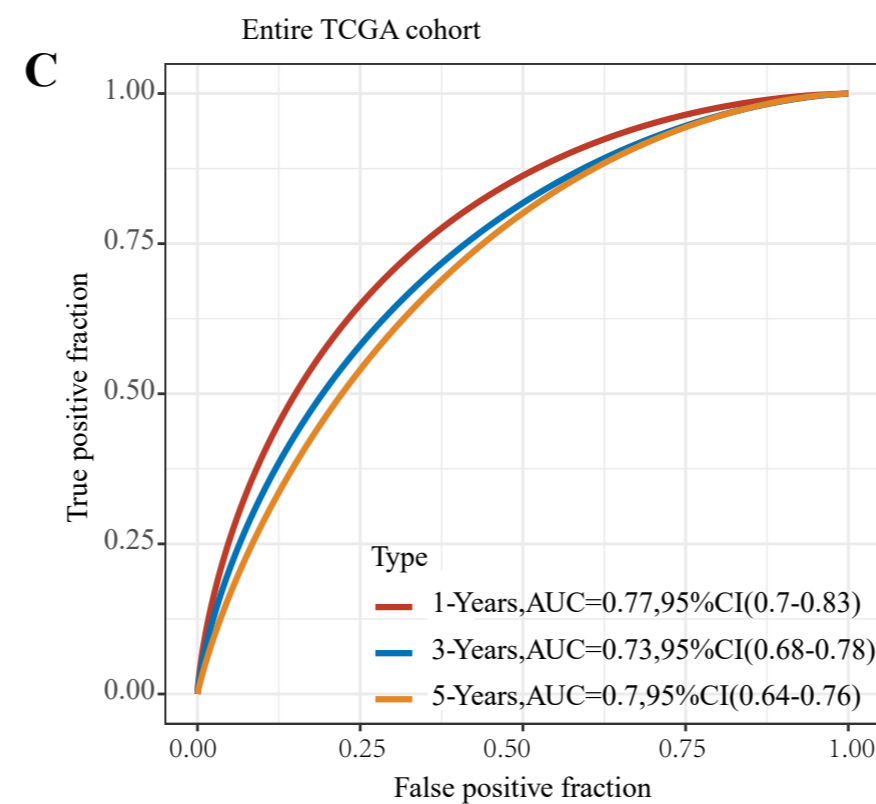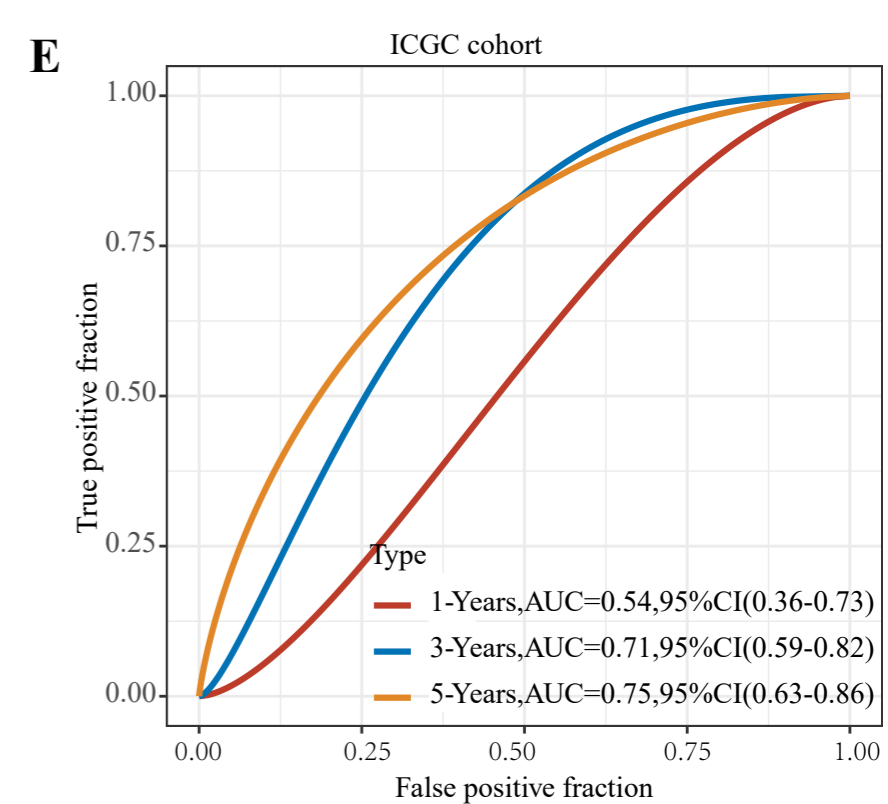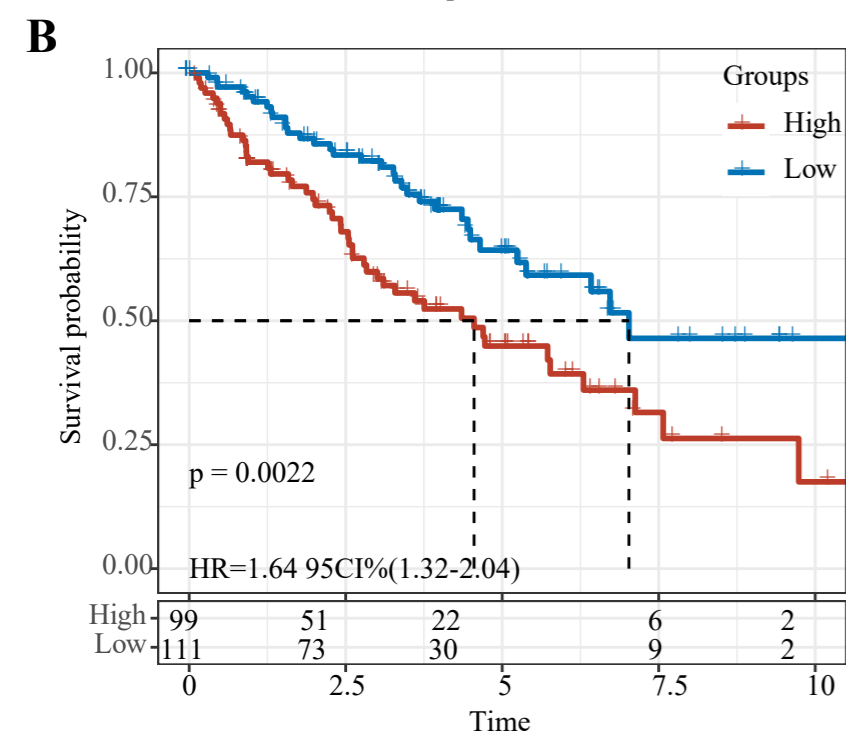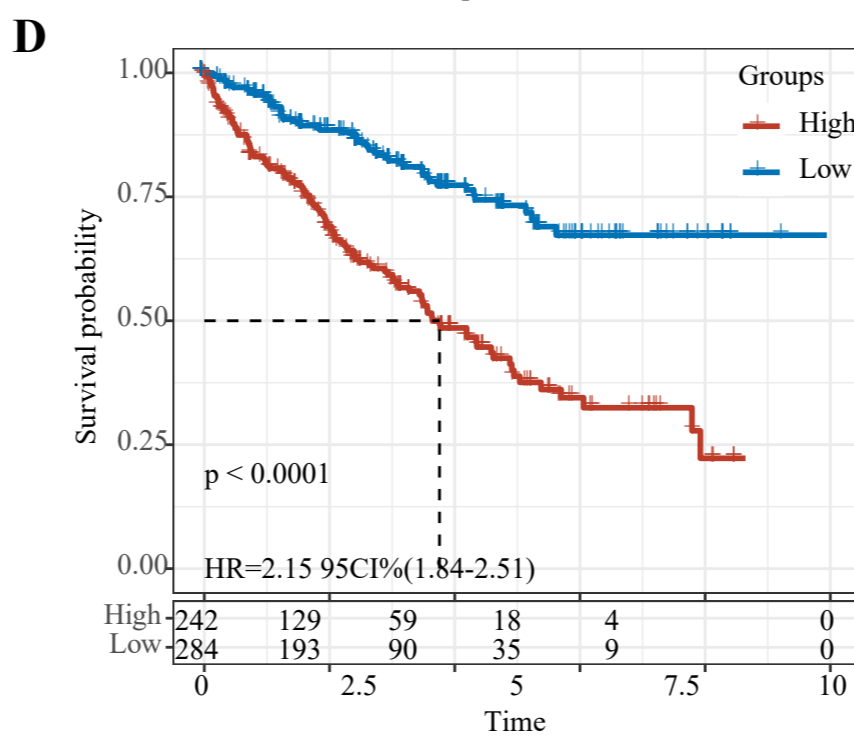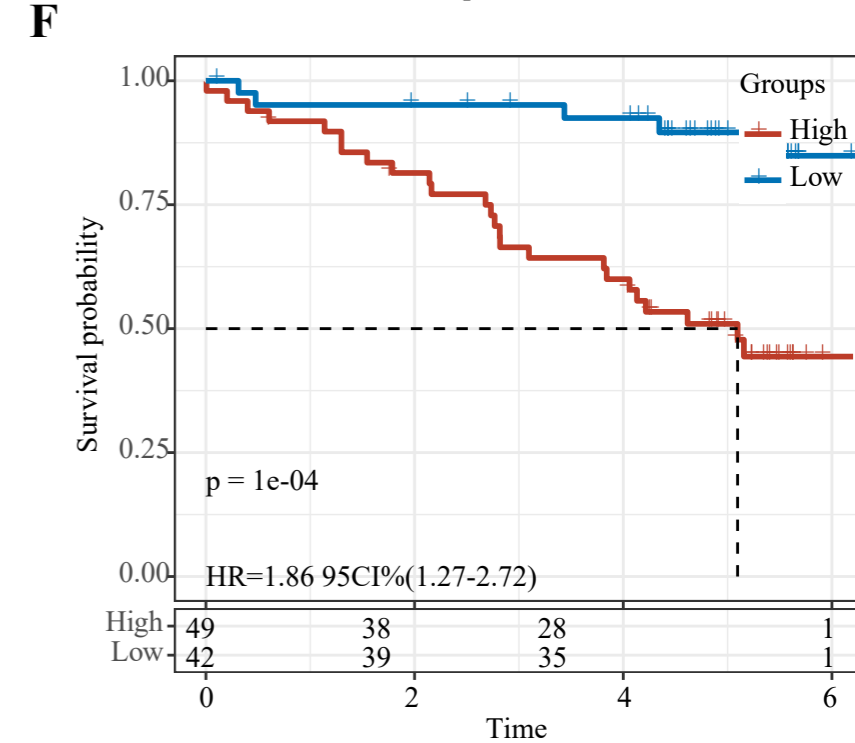

Supplement: Supplementary Figure 2 — Comparison of TIDE score, T cell dysfunction score and T cell rejection score of molecular subtypes. (A) In the TCGA data set, TIDE scores are different in the three molecular subtypes. (B) In the TCGA data set, T cell dysfunction scores are different in the three molecular subtypes. (C) In the TCGA data set, T cell exclusion scores are different in the three molecular subtypes. [file DataSheet_2.pdf]

A

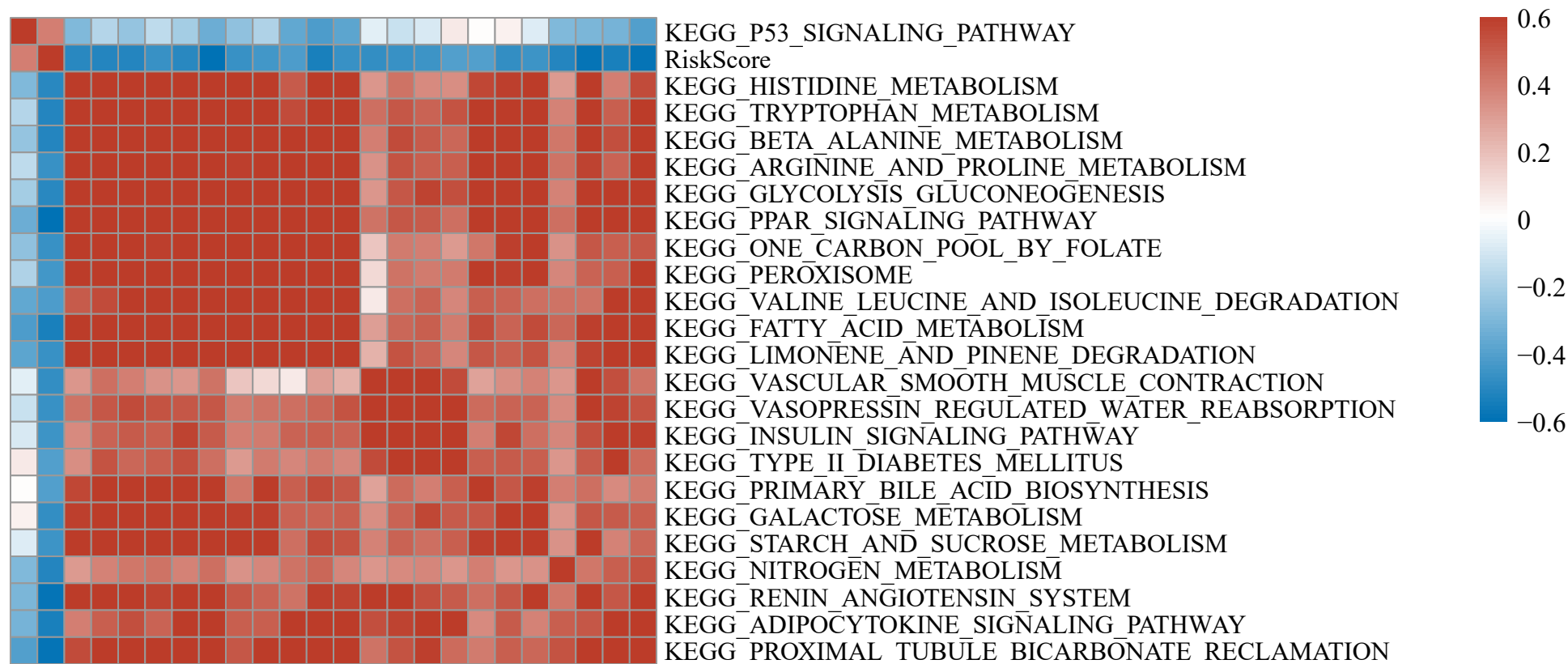

B

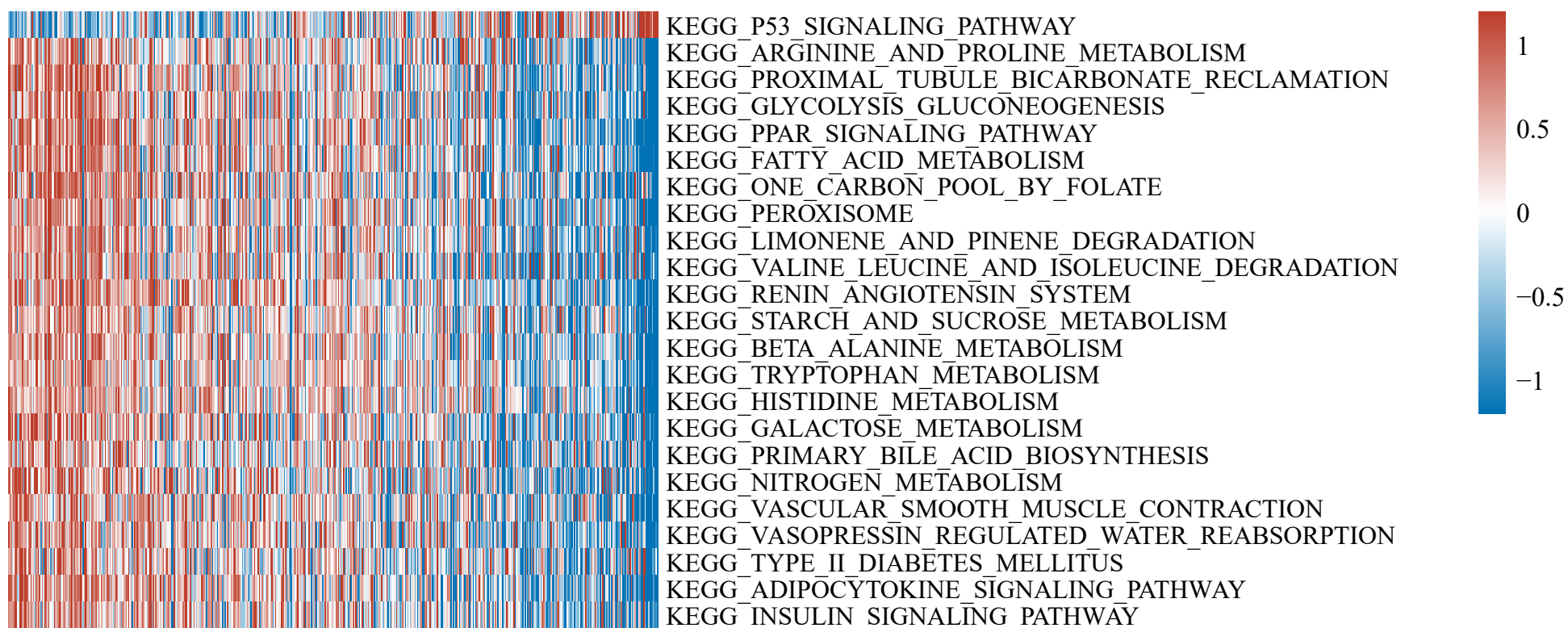

Supplement: Supplementary Figure 3 — (A) ROC curve and AUC of the 8-gene signature in TCGA test set; (B) KM survival curve of 8-gene signature in TCGA test set. (C) ROC curve and AUC of 8-gene signature in TCGA entire data set; (D) KM survival curve of 8-gene signature in TCGA entire data set. (E) ROC curve and AUC of 8-gene signature in the ICGC cohort; (F) KM survival curve of 8-gene signature in the ICGC cohort. [file DataSheet_3.pdf]

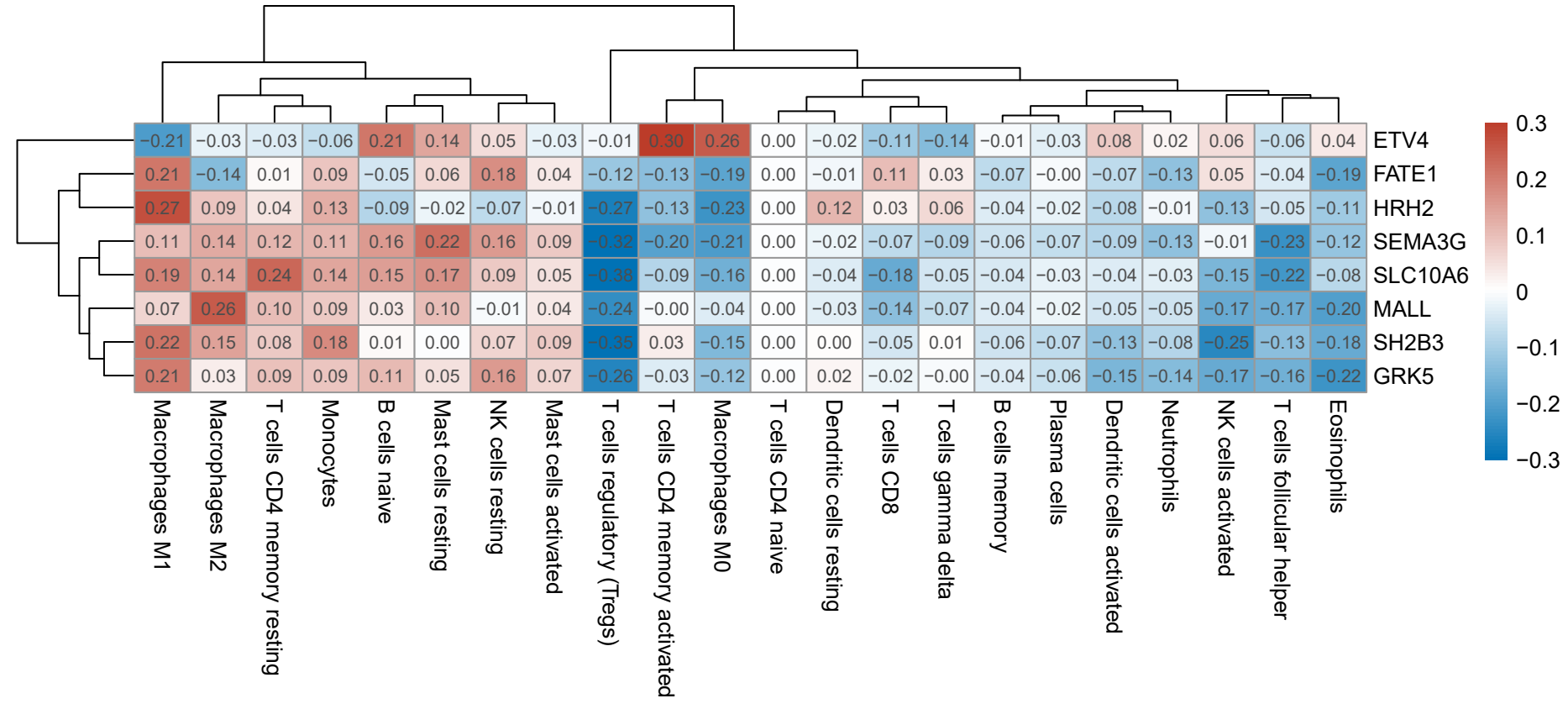

Supplement: Supplementary Figure 4 — The relationship between RiskScore and the pathways. (A) The correlation coefficient clustering of KEGG pathways greater than 0.4 and the RiskScore; (B) ssGSEA scores of KEGG pathways with a correlation greater than 0.4 in each sample as the RiskScore increases, the horizontal axis represents the sample, and the Riskscore increases from left to right. [file DataSheet_4.pdf]

A

TCGA

category 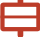 C1 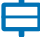 C2 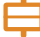 C3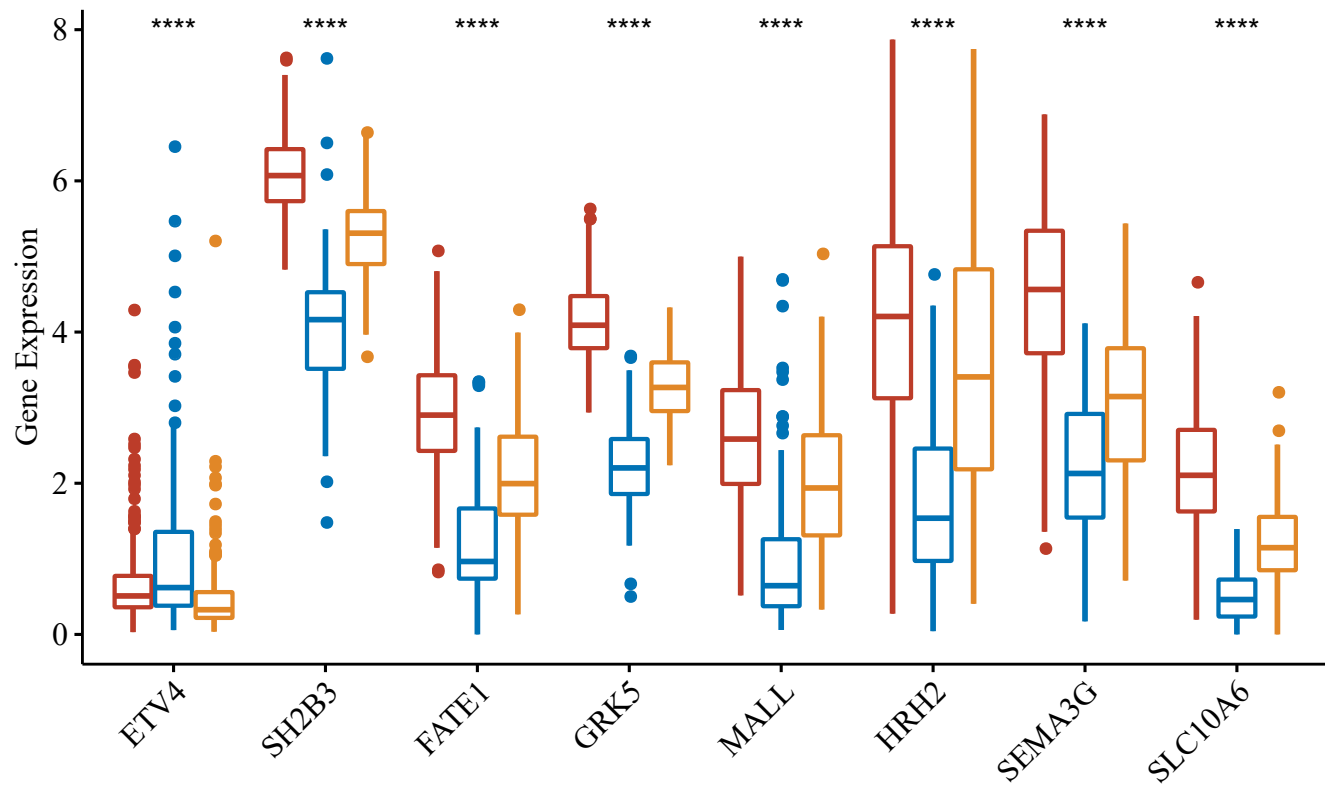

B

ICGC

category 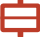 C1 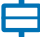 C2 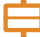 C3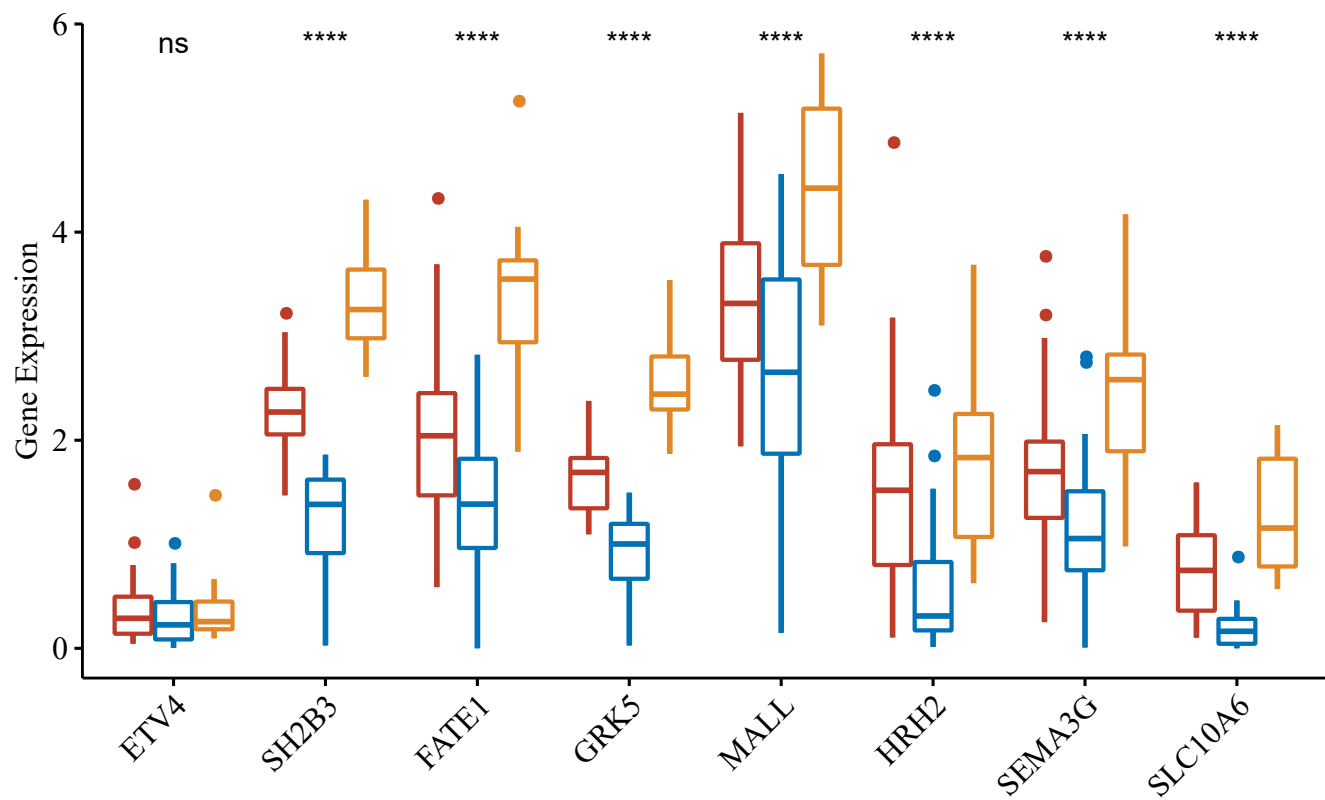

Supplement: Supplementary Figure 5 — The PPI analysis for the eight prognostic genes. [file DataSheet_5.pdf]

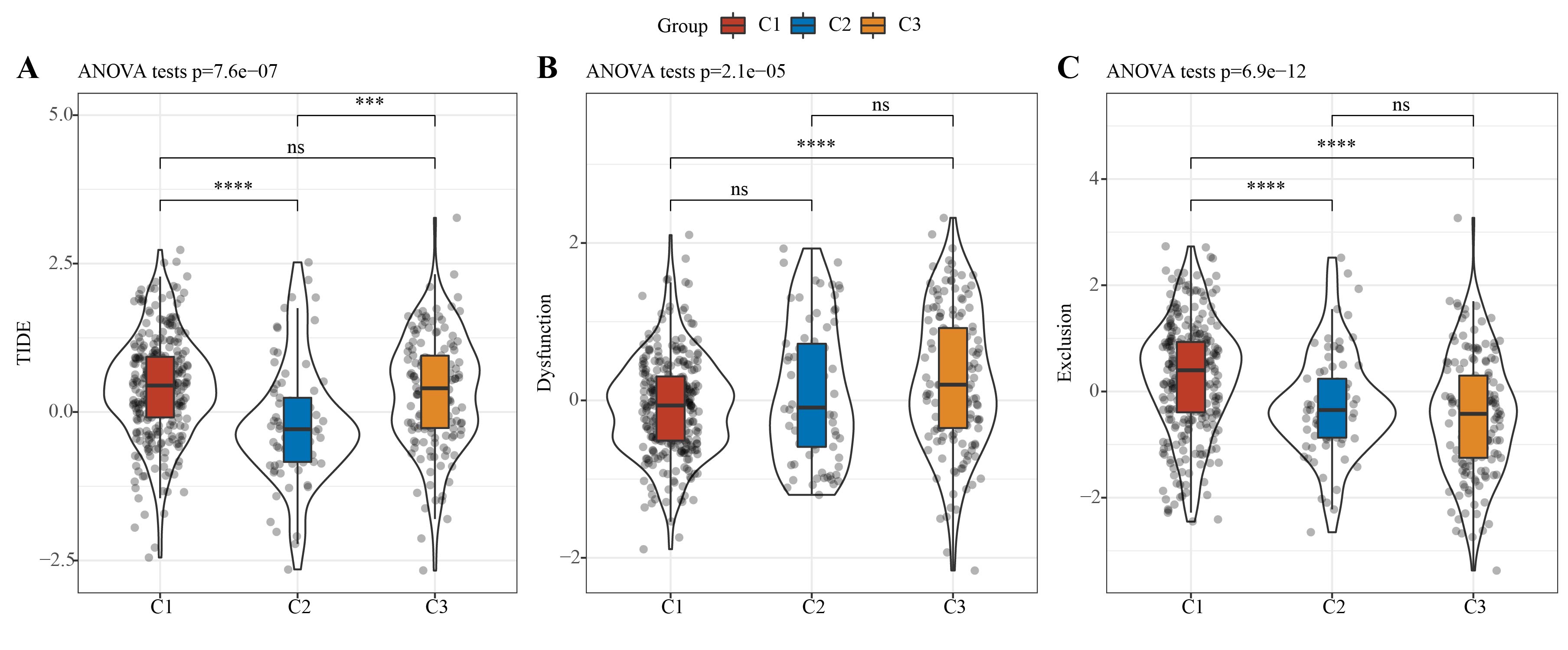

Supplement: Supplementary Figure 7 — The expression of eight prognostic genes in three molecular subtypes in TCGA (A) and ICGC (B) datasets. Kruskal-Wallis test was performed. ns, no significance. ****P < 0.0001. [file Image_1.jpeg]

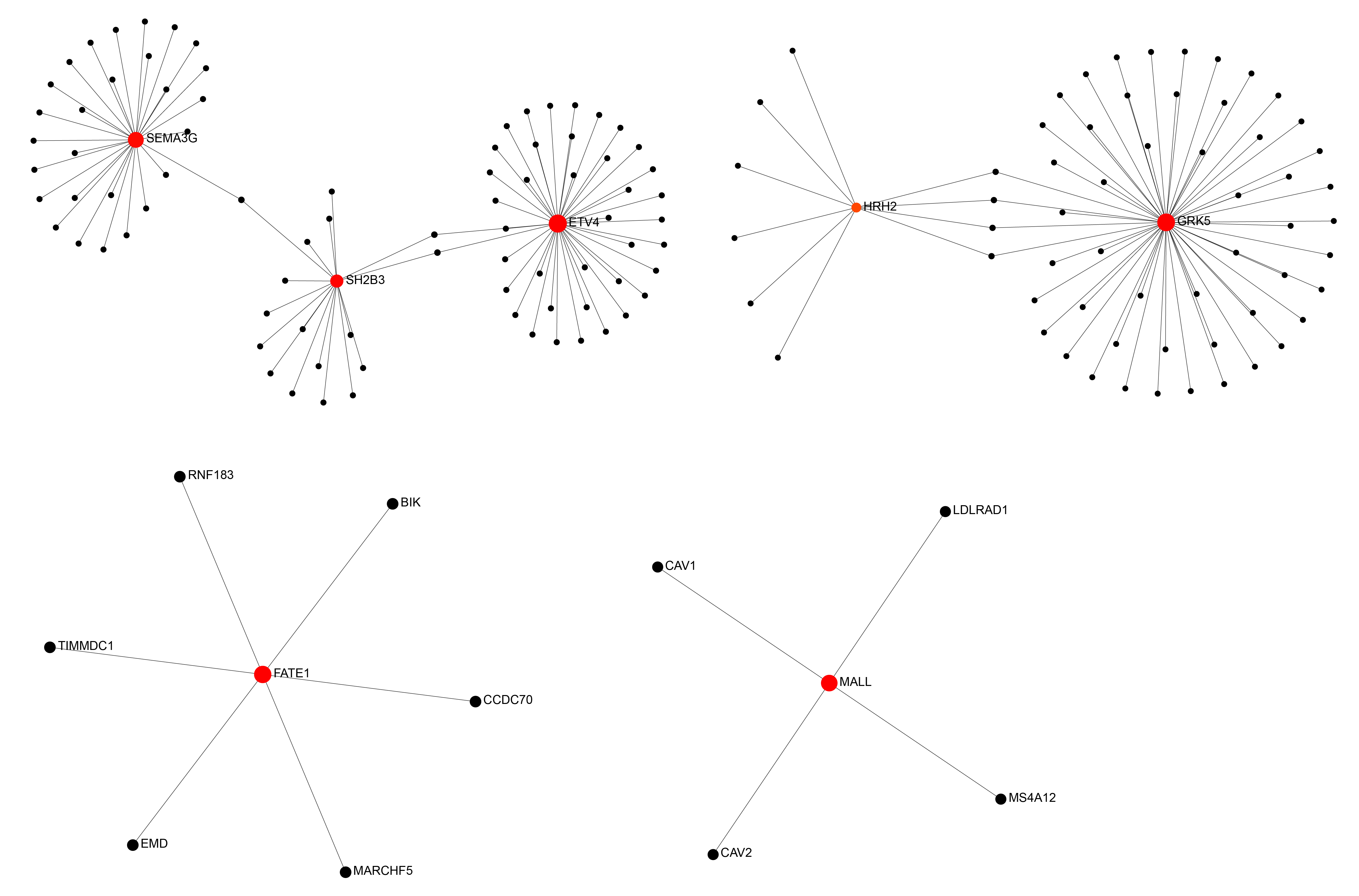

Supplement: Supplementary Figure 8 — Single-factor and multi-factor cox analyses on the 8-gene signature(A) Single-factor analysis of TCGA entire data set; (B) Multi-factor analysis of TCGA entire data set. (C) Nomogram constructed with clinical characteristics and RiskScore; (D) Correction chart of survival rate of nomogram; (E) DCA chart. [file Image_2.jpeg]
