# Supplementary material for: Identification of epigenetic dysregulation gene markers and immune landscape in kidney renal clear cell carcinoma by comprehensive genomic analysis
Source: Front Immunol. 2022 Aug 18;13:901662. doi: 10.3389/fimmu.2022.901662 (PMC9433776; doi:10.3389/fimmu.2022.901662)
Supplement: Supplementary Figure 6 — Pearson correlation analysis between the eight prognostic genes and the ssGSEA score of immune cells. Red and blue indicates positive and negative correlations respectively. [file DataSheet_6.pdf]

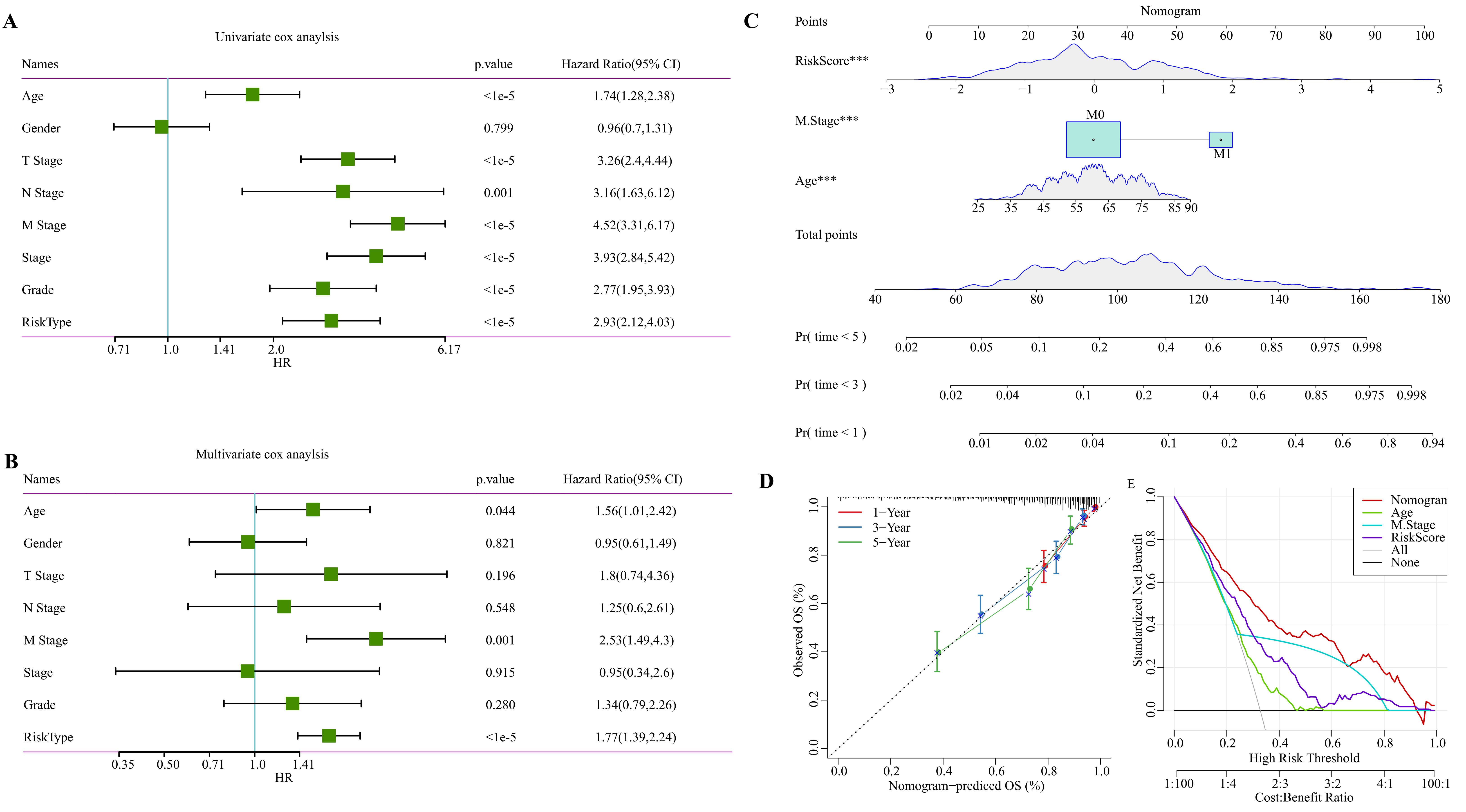

A
Univariate cox anaylsis
Names
p.value
Hazard Ratio(95% CI)
Age
<1e-5
1.74(1.28,2.38)
Gender
0.799
0.96(0.7,1.31)
T Stage
<1e-5
3.26(2.4,4.44)
N Stage
0.001
3.16(1.63,6.12)
M Stage
<1e-5
4.52(3.31,6.17)
Stage
<1e-5
3.93(2.84,5.42)
Grade
<1e-5
2.77(1.95,3.93)
RiskType
<1e-5
2.93(2.12,4.03)
0.71
1.0
1.41
2.0
6.17
HR
B
Multivariate cox anaylsis
Names
p.value
Hazard Ratio(95% CI)
Age
0.044
1.56(1.01,2.42)
Gender
0.821
0.95(0.61,1.49)
T Stage
0.196
1.8(0.74,4.36)
N Stage
0.548
1.25(0.6,2.61)
M Stage
0.001
2.53(1.49,4.3)
Stage
0.915
0.95(0.34,2.6)
Grade
0.280
1.34(0.79,2.26)
RiskType
<1e-5
1.77(1.39,2.24)
0.35
0.50
0.71
1.0
1.41
HR
C
Nomogram
Points
RiskScore***
M.Stage***
M0
M1
Age***
Total points
Pr(time < 5)
Pr(time < 3)
Pr(time < 1)
D
1−Year
3−Year
5−Year
Observed OS (%)
Nomogram−prediced OS (%)
E
Nomogram
Age
M.Stage
RiskScore
All
None
Standardized Net Benefit
High Risk Threshold
Cost:Benefit Ratio
1:100
1:4
2:3
3:2
4:1
100:1
