# Supplementary material for: Identification of epigenetic dysregulation gene markers and immune landscape in kidney renal clear cell carcinoma by comprehensive genomic analysis
Source: Front Immunol. 2022 Aug 18;13:901662. doi: 10.3389/fimmu.2022.901662 (PMC9433776; doi:10.3389/fimmu.2022.901662)
Supplement: Supplementary file 9 [file Table_1.docx]

Supplementary Table 1. The primer sequences utilized in qRT-PCR.

| Primer name | Sequence (5’-3’) |
| --- | --- |
| ETV4-F | 5'- GCTCGCTGAAGCTCAGGT -3' |
| ETV4-R | 5'- TCCTTCTTGATCCTGGTGGT -3' |
| SH2B3-F | 5'- CCCACCTTAGTTCTTCTG -3' |
| SH2B3-R | 5'- GGATGAGTCCATTTCGTA -3' |
| FATE1-F | 5'- GGCAATTTCCAAGGCATACG -3' |
| FATE1-R | 5'- CTAGTCTGCGCCACTGCATC -3' |
| GRK5-F | 5'- CAATCGGGAGGCTGCTTTTC -3' |
| GRK5-R | 5'- TCCTTCCCTTTCTCTCCCAG -3' |
| MALL-F | 5'- CAGCCTCGTTCTTCGC -3' |
| MALL-R | 5'- TTCCGTTTGTCATCCA -3' |
| HRH2-F | 5'- ACCAGCAAGGGCAATCATAC -3' |
| HRH2-R | 5'- CATGATCAGTAGCGGGAGGT -3' |
| SEMA3G-F | 5'- TGTATGCTATTAACTCCTGGAA -3' |
| SEMA3G-R | 5'- ACAATACACAGATACACAGTAAT -3' |
| SLC10A6-F | 5'- TATGACAACCTGTTCCACCG -3' |
| SLC10A6-R | 5'- GAATGGTCAGGCACACAAGG -3' |
| GAPDH-F | 5'- TGCACCACCAACTGCTTAGC -3' |
| GAPDH-R | 5'- GGCATGGACTGTGGTCATGAG -3' |
